# Supplementary material for: Diabetic sensory neuropathy and insulin resistance are induced by loss of UCHL1 in Drosophila
Source: Nat Commun. 2024 Jan 11;15:468. doi: 10.1038/s41467-024-44747-9 (PMC10784524; doi:10.1038/s41467-024-44747-9)
Supplement: Supplementary file 1 — Supplementary Information [file 41467_2024_44747_MOESM1_ESM.pdf]

Supplemental Information

Diabetic sensory neuropathy and insulin resistance are induced by loss of *UCHL1* in *Drosophila*

Daewon Lee, Eunju Yoon, Su Jin Ham, Kunwoo Lee, Hansaem Jang, Daihn Woo, Da Hyun Lee, Sehyeon Kim, Sekyu Choi, and Jongkyeong Chung

*corresponding author. Email: sekyuchoi@postech.ac.kr; jkc@snu.ac.kr

This information includes:

Supplementary Figures 1-12

Supplementary Tables 1-2

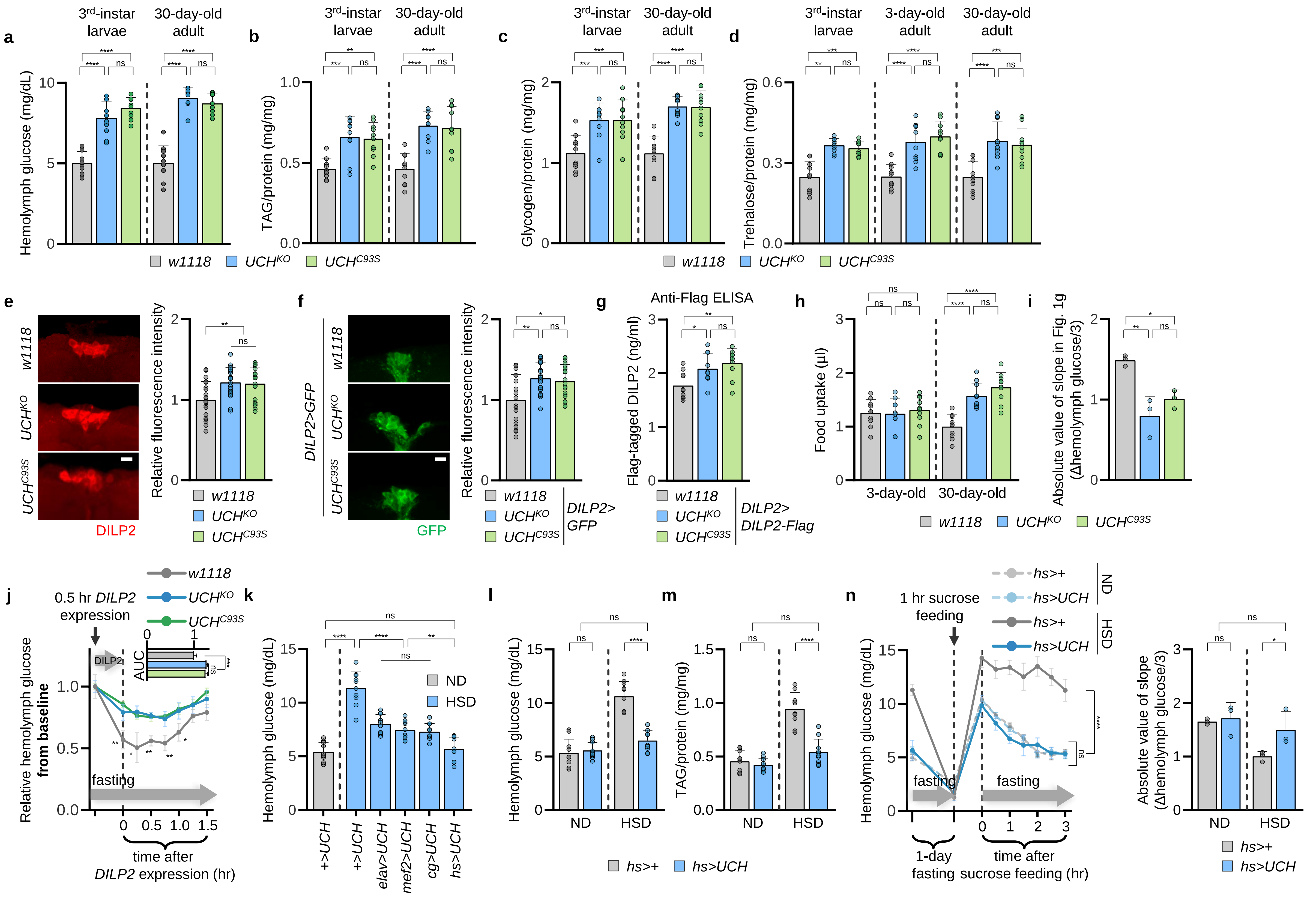
a
3rd-instar larvae
30-day-old adult
Hemolymph glucose (mg/dL)
b
TAG/protein (mg/mg)
c
Glycogen/protein (mg/mg)
d
3-day-old adult
Trehalose/protein (mg/mg)
w1118
UCHKO
UCHC93S
e
DILP2
Relative fluorescence intensity
f
DILP2>GFP
GFP
DILP2> GFP
g
Anti-Flag ELISA
Flag-tagged DILP2 (ng/ml)
DILP2> DILP2-Flag
h
Food uptake (µl)
3-day-old
30-day-old
i
Absolute value of slope in Fig. 1g (Δhemolymph glucose/3)
j
0.5 hr DILP2 expression
DILP2
AUC
Relative hemolymph glucose from baseline
fasting
time after DILP2 expression (hr)
k
ND
HSD
+>UCH
elav>UCH
mef2>UCH
cg>UCH
hs>UCH
l
m
TAG/protein (mg/mg)
hs>+
hs>UCH
n
1 hr sucrose feeding
1-day fasting
time after sucrose feeding (hr)
Absolute value of slope (Δhemolymph glucose/3)

Supplementary Fig. 1 | T2D-like phenotypes are induced by *UCHL1* mutations and T2D-related defects caused by HSD are improved by *UCHL1* overexpression. a, Glucose concentrations in the hemolymph of 3rd instar larvae and 30-day-old flies. n = 10. **b,** TAG concentrations per total body protein of 3rd instar larvae and 30-day-old flies. n = 10. **c,** Glycogen concentrations per total body protein of 3rd instar larvae and 30-day-old flies. n = 10. **d,** Trehalose concentrations per total body protein of 3rd instar larvae, 3-day-old, and 30-day-old flies. n = 10. **e,** Left, confocal immunofluorescence images of the brain stained with anti-DILP2 antibody. Right, the relative fluorescence intensity of DILP2 staining normalized to the intensity of *w1118*. n = 20. Scale bar, 10 μm. **f,** Left, confocal fluorescence images of the brain expressing *DILP2>GFP*. Right, the relative fluorescence intensity of GFP normalized to the intensity of control flies. n = 20. Scale bar, 10 μm. **g,** Measuring DILP2-Flag concentration using DILP2-Flag ELISA. n = 10. **h,** Measuring food uptake of 3-day-old and 30-day-old flies using CAFE assay. n = 10. **i,** Absolute value of the slope of Fig. 1g from right after the sucrose feeding to 3 hours after the sucrose feeding (|(hemolymph glucose at 3 hours after sucrose feeding – hemolymph glucose at 0 hours after sucrose feeding)/ 3|). n = 3. **j**, Relative levels of hemolymph glucose normalized to the glucose levels of each genotype before DILP2 expression (from baseline) depicted in Fig. 1h. The bar graph at the upper side represents the area under curve (AUC) of the line graph in the same panel. n = 3. **k,** Glucose concentrations in the hemolymph of 3-day-old flies upon ND or HSD. n = 10. **l,** Glucose concentrations in the hemolymph of 3-day-old flies upon ND or HSD. n = 10. **m,** TAG concentrations per total body protein of 3-day-old flies upon ND or HSD. n = 10. **n,** Left, glucose concentrations in the hemolymph of the flies. Each 3-day-old fly feeding ND or HSD continuously after eclosion was on 10% sucrose solution for 1 hour after 1-day fasting. After the 1-hour sucrose feeding, the hemolymph glucose levels were measured every 30 minutes while fasting the flies again. The indicated statistical significance was calculated from the data after the 1-hour feeding. n = 3. Right, absolute value of the slope of left graph from right after the sucrose feeding to 3 hours after the sucrose feeding (|(hemolymph glucose at 3 hours after sucrose feeding – hemolymph glucose at 0 hours after sucrose feeding)/ 3|). n = 3. ND, normal diet. HSD, high-sucrose diet. Data are presented as mean ± SD. One-way ANOVA with Tukey's multiple comparison test was used (**a**, **b**, **c**, **d**, **e**, **f**, **g**, **h**, **i**, **j**, and **k**). Two-way ANOVA with Sidak's multiple comparison test was used (**l**, **m**, and left and right graph of **n**). *p < 0.05. **p < 0.01. ***p < 0.001. ****p < 0.0001. ns, no significant.

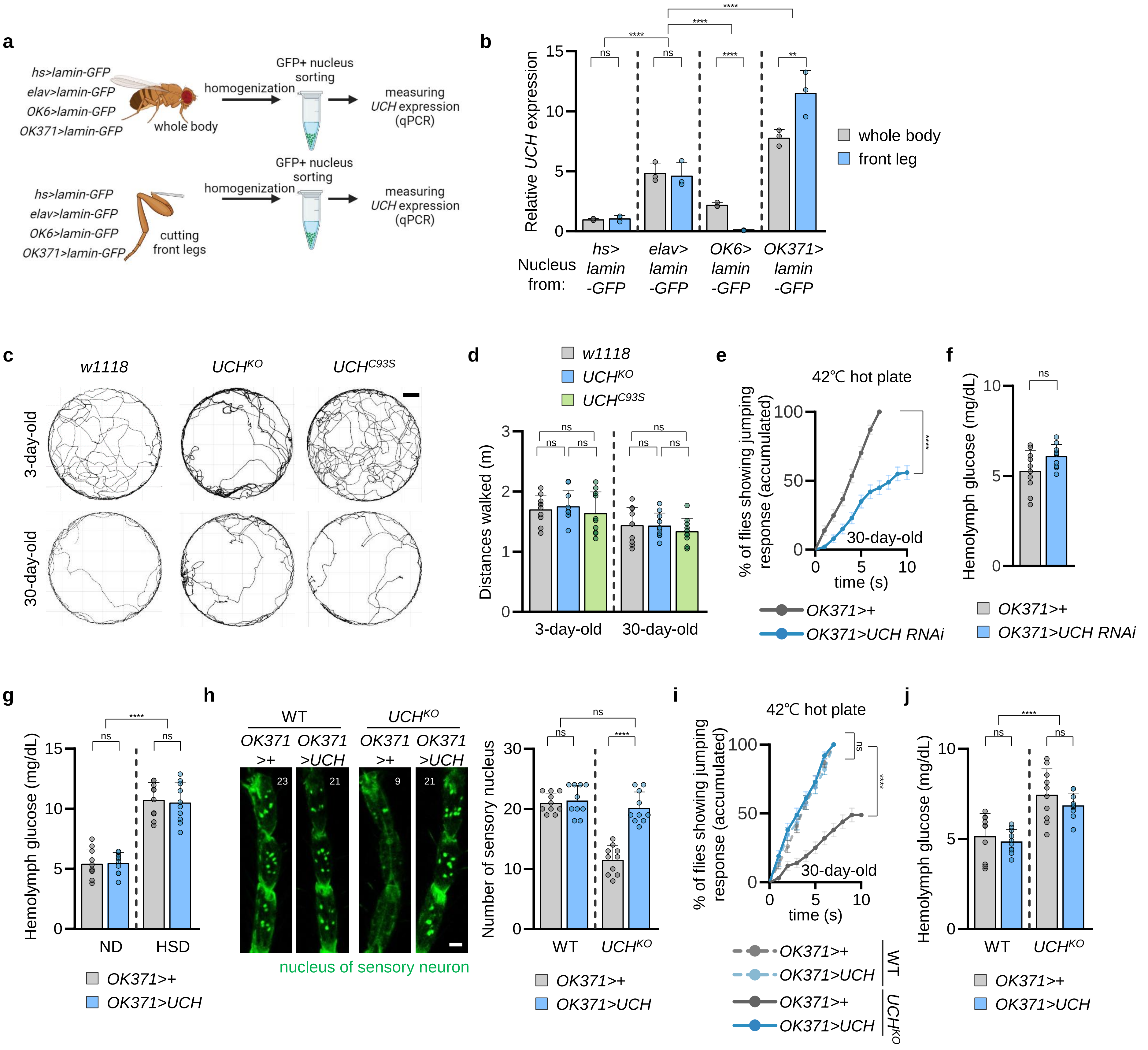
a
hs>lamin-GFP
elav>lamin-GFP
OK6>lamin-GFP
OK371>lamin-GFP
whole body
homogenization
GFP+ nucleus sorting
measuring UCH expression (qPCR)
cutting front legs
b
Relative UCH expression
Nucleus from:
hs> lamin -GFP
elav> lamin -GFP
OK6> lamin -GFP
OK371> lamin -GFP
whole body
front leg
c
w1118
UCH^KO
UCH^C93S
3-day-old
30-day-old
d
Distances walked (m)
3-day-old
30-day-old
e
42℃ hot plate
% of flies showing jumping response (accumulated)
30-day-old
time (s)
OK371>+
OK371>UCH RNAi
f
Hemolymph glucose (mg/dL)
OK371>+
OK371>UCH RNAi
g
Hemolymph glucose (mg/dL)
ND
HSD
OK371>+
OK371>UCH
h
WT
UCH^KO
OK371 >+
OK371 >UCH
nucleus of sensory neuron
Number of sensory nucleus
OK371>+
OK371>UCH
i
42℃ hot plate
% of flies showing jumping response (accumulated)
30-day-old
time (s)
OK371>+
OK371>UCH
WT
UCH^KO
j
Hemolymph glucose (mg/dL)
WT
UCH^KO
OK371>+
OK371>UCH

Supplementary Fig. 2 | Autonomous degeneration of sensory neurons is induced by *UCHL1* mutations. a, Schematic diagram of the experiments for measuring *UCH* expression from extracted nuclei. GFP-positive (GFP^{+}) nuclei were sorted out from each genotype of the flies' whole bodies or front legs, and the levels of *UCH* expression were measured by qPCR. *hs>lamin-GFP*, *elav>lamin-GFP*, *OK6>lamin-GFP*, and *OK371>lamin-GFP* were generated to label GFP at the nuclei of whole tissues, neurons, motor neurons, and sensory neurons, respectively. The illustration was created using Biorender.com. **b,** Relative expressions of *UCH* normalized to *rp49* expression from extracted GFP^{+} nuclei of the flies' whole bodies or front legs. n = 3. **c,** Representative trajectories of 3-day-old and 30-day-old flies. Scale bar, 10 mm. **d,** Measurement of the distances 3-day-old and 30-day-old flies walked. n = 10. **e,** Cumulative percentage of 30-day-old flies showing escape responses on the 42°C hot plates within 10 seconds. n = 100. **f,** Glucose concentrations in the hemolymph of 30-day-old flies. n = 10. **g,** Glucose concentrations in the hemolymph of 30-day-old flies upon ND or HSD. n = 10. **h,** Left, confocal fluorescence images of tarsal segments 3, 4, and 5 at the front legs of 30-day-old flies expressing *OK371>nlsGFP*. The number in panels indicates the number of green signals in each image. Respective images were obtained from one of the left or right front legs. Green, the nucleus of sensory neuron. Scale bar, 20 μm. Right, the numbers of green signals at the tarsal segments 3, 4, and 5 of the front legs of 30-day-old flies expressing *OK371>nlsGFP*. n = 10. **i,** Cumulative percentage of 30-day-old flies showing escape responses on the 42°C hot plates within 10 seconds. n = 100. **j,** Glucose concentrations in the hemolymph of 3-day-old flies. n = 10. ND, normal diet. HSD, high-sucrose diet. Data are presented as mean ± SD. Two-way ANOVA with Sidak's multiple comparison test was used (**b**, **g**, **h**, and **j**). One-way ANOVA with Tukey's multiple comparison test was used (**d**). Mantel-Cox test was used (**e** and **i**). Two-tailed paired Student's t-test was used (**f**). **p < 0.01, ****p < 0.0001. ns, no significant.

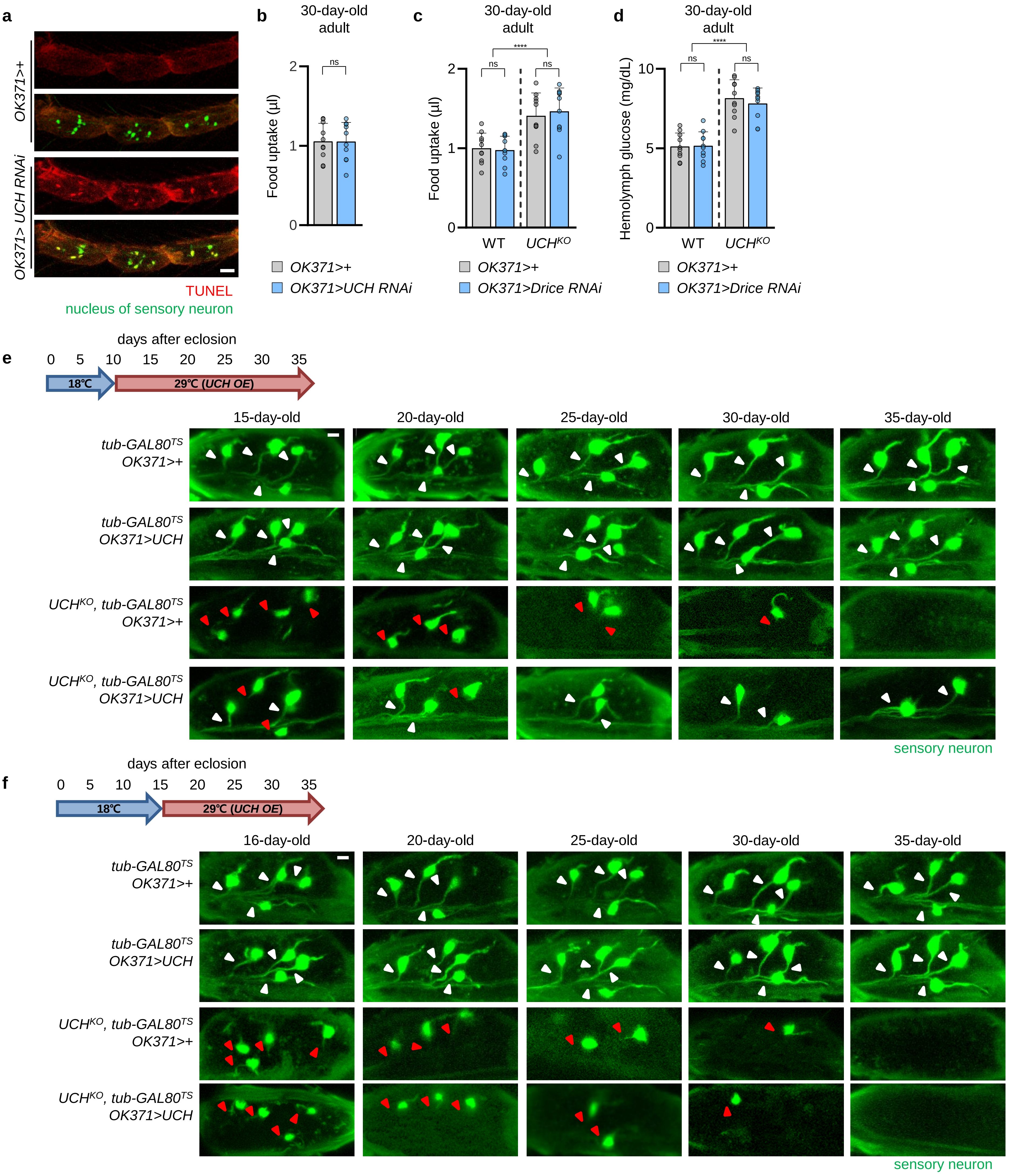
a
OK371>+
OK371> UCH RNAi
TUNEL
nucleus of sensory neuron
b
30-day-old adult
Food uptake (µl)
ns
OK371>+
OK371>UCH RNAi
c
30-day-old adult
Food uptake (µl)
ns
****
WT
UCHKO
OK371>+
OK371>Drice RNAi
d
30-day-old adult
Hemolymph glucose (mg/dL)
ns
****
WT
UCHKO
OK371>+
OK371>Drice RNAi
e
days after eclosion
0 5 10 15 20 25 30 35
18℃
29℃ (UCH OE)
15-day-old
20-day-old
25-day-old
30-day-old
35-day-old
tub-GAL80TS OK371>+
tub-GAL80TS OK371>UCH
UCHKO, tub-GAL80TS OK371>+
UCHKO, tub-GAL80TS OK371>UCH
sensory neuron
f
days after eclosion
0 5 10 15 20 25 30 35
18℃
29℃ (UCH OE)
16-day-old
20-day-old
25-day-old
30-day-old
35-day-old
tub-GAL80TS OK371>+
tub-GAL80TS OK371>UCH
UCHKO, tub-GAL80TS OK371>+
UCHKO, tub-GAL80TS OK371>UCH
sensory neuron

Supplementary Fig. 3 | Apoptosis of sensory neurons did not affect the feeding behavior or hemolymph glucose level, and the loss of sensory neurons only occur in those with impaired axons. a, Confocal fluorescence images for TUNEL assays of tarsal segments 3, 4, and 5 at the front legs of flies expressing *OK371>nlsGFP*. Respective images were obtained from one of the left or right front legs. Red, TUNEL signal. Green, the nucleus of sensory neuron. Scale bar, 20 μm. **b,** Measuring food uptake of 30-day-old flies using CAFE assay. n = 10. **c,** Measuring food uptake of 30-day-old flies using CAFE assay. n = 10. **d,** Glucose concentrations in the hemolymph of 30-day-old flies. n = 10. **e,** Confocal fluorescence images for axons and somas of sensory neurons of tarsal segments 4 at the front legs of flies expressing *OK371>GFP* at 15, 20, 25, 30, and 35 days of age. Starting from 10 days of age, the flies were placed at 29°C to induce continuous exogenous expression of *UCH*. Green, sensory neuron. White or red triangles indicate intact or impaired axons, respectively. Scale bar, 10 μm. **f,** Confocal fluorescence images for axons and somas of sensory neurons of tarsal segments 4 at the front legs of flies expressing *OK371>GFP* at 16, 20, 25, 30, and 35 days of age. Starting from 15 days of age, the flies were placed at 29°C to induce continuous exogenous expression of *UCH*. Sensory neurons were observed at 16 days of age owing to the GFP induction for 1 day. Green, sensory neuron. White or red triangles indicate intact or impaired axons, respectively. Scale bar, 10 μm. Data are presented as mean ± SD. Two-tailed paired Student's t-test was used (**b**). Two-way ANOVA with Sidak's multiple comparison test was used (**c** and **d**). ****p < 0.0001. ns, no significant.

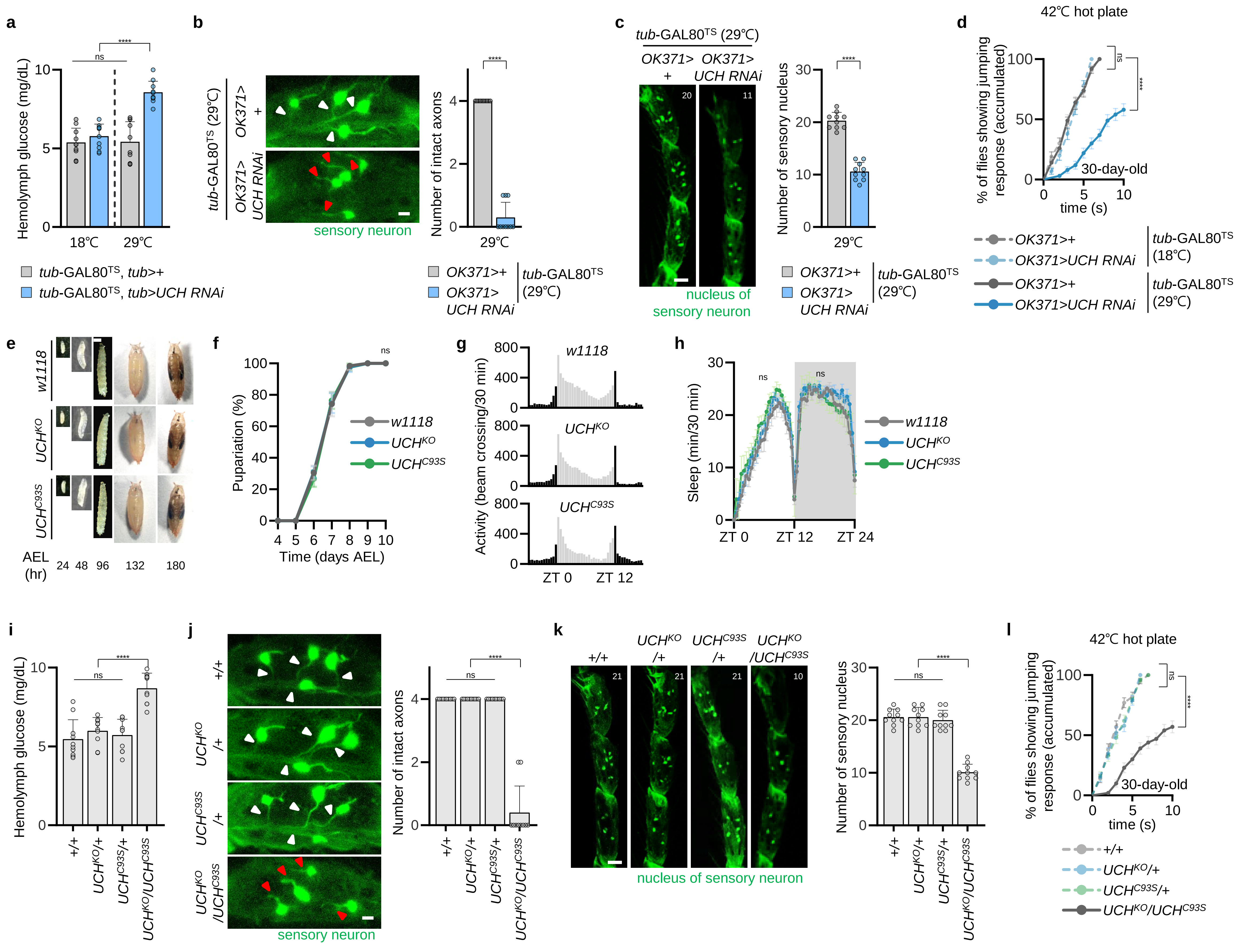

a
Hemolymph glucose (mg/dL)
18℃
29℃
ns
****
tub-GAL80TS, tub>+
tub-GAL80TS, tub>UCH RNAi
b
tub-GAL80TS (29℃)
OK371> +
OK371> UCH RNAi
sensory neuron
Number of intact axons
29℃
OK371>+
OK371> UCH RNAi
tub-GAL80TS (29℃)
c
tub-GAL80TS (29℃)
OK371> +
OK371> UCH RNAi
nucleus of sensory neuron
Number of sensory nucleus
29℃
d
42℃ hot plate
% of flies showing jumping response (accumulated)
time (s)
30-day-old
OK371>+
OK371>UCH RNAi
tub-GAL80TS (18℃)
tub-GAL80TS (29℃)
e
w1118
UCHKO
UCHC93S
AEL (hr)
24 48 96 132 180
f
Pupariation (%)
Time (days AEL)
w1118
UCHKO
UCHC93S
g
Activity (beam crossing/30 min)
ZT 0
ZT 12
h
Sleep (min/30 min)
ZT 0 ZT 12 ZT 24
i
Hemolymph glucose (mg/dL)
+/+
UCHKO/+
UCHC93S/+
UCHKO/UCHC93S
j
sensory neuron
Number of intact axons
k
nucleus of sensory neuron
Number of sensory nucleus
l
42℃ hot plate
% of flies showing jumping response (accumulated)
time (s)
30-day-old

Supplementary Fig. 4 | The T2D- or DSN-like phenotypes of *UCH* mutants are not caused by mechanisms other than insulin resistance. a, Glucose concentrations in the hemolymph glucose of 3-day-old flies. Flies of indicated genotypes were raised at 18℃ until eclosion. Following eclosion, they were either kept at 18℃ or transferred to 29℃ for a period of 3 days before hemolymph was extracted. n = 10. **b,** Left, confocal fluorescence images for axons and somas of sensory neurons of tarsal segments 4 at the front legs of 15-day-old flies expressing *OK371>GFP*. Respective images were obtained from one of the left or right front legs. Green, sensory neuron. White or red triangles indicate intact or impaired axons, respectively. Scale bar, 10 μm. Right, the number of intact axons at tarsal segments 4 at the front legs of 15-day-old flies expressing *OK371>GFP*. Flies of indicated genotypes were raised at 18℃ until eclosion. Following eclosion, they were transferred to 29℃ for a period of 15 days before the leg images were obtained. n = 10. **c,** Left, confocal fluorescence images of tarsal segments 3, 4, and 5 at the front legs of 30-day-old flies expressing *OK371>nlsGFP*. The number in panels indicates the number of green signals in each image. Respective images were obtained from one of the left or right front legs. Green, the nucleus of sensory neuron. Scale bar, 20 μm. Right, the numbers of green signals at the tarsal segments 3, 4, and 5 of the front legs of 30-day-old flies expressing *OK371>nlsGFP*. Flies of indicated genotypes were raised at 18℃ until eclosion. Following eclosion, they were transferred to 29℃ for a period of 30 days before the leg images were obtained. n = 10. **d,** Cumulative percentage of 30-day-old flies showing escape responses on the 42℃ hot plates within 10 seconds. Flies of indicated genotypes were raised at 18℃ until eclosion. Following eclosion, they were either kept at 18℃ or transferred to 29℃ for a period of 30 days before the experiments were conducted. n = 100. **e,** Images of larvae with indicated genotypes at 24, 48, 96, 132, and 180 hours after egg laying (AEL). Scale bar, 0.5 mm. **f,** Percentage of pupariation at each time point. n = 100. **g,** Daily activity profiles of adult flies. n = 15. **h,** Sleep traces of adult flies. n = 15. **i,** Glucose concentrations in the hemolymph glucose of 3-day-old flies. n = 10. **j,** Left, confocal fluorescence images for axons and somas of sensory neurons of tarsal segments 4 at the front legs of 15-day-old flies expressing *OK371>GFP*. Respective images were obtained from one of the left or right front legs. Green, sensory neuron. White or red triangles indicate intact or impaired axons, respectively. Scale bar, 10 μm. Right, the number of intact axons at tarsal segments 4 at the front legs of 15-day-old flies expressing *OK371>GFP*. n = 10. **k,** Left, confocal fluorescence images of tarsal segments 3, 4, and 5 at the front legs of 30-day-old flies expressing *OK371>nlsGFP*. The number in panels indicates the number of green signals in each image. Respective images were obtained from one of the left or right front legs. Green, the nucleus of sensory neuron. Scale bar, 20 μm. Right, the numbers of green signals at the tarsal segments 3, 4, and 5 of the front legs of 30-day-old flies expressing *OK371>nlsGFP*. n = 10. **l,** Cumulative percentage of 30-day-old flies showing escape responses on the 42℃ hot plates within 10 seconds. n = 100. ZT, Zeitgeber time. Data are presented as mean ± SD. Two-way ANOVA with Sidak's multiple comparison test was used (**a**). Two-tailed paired Student's t-test was used (**b** and **c**). Mantel-Cox test was used (**d** and **l**). One-way ANOVA with Tukey's multiple comparison test was used (**f**, **h**, **i**, **j**, and **k**). ****$p < 0.0001$. ns, no significant.

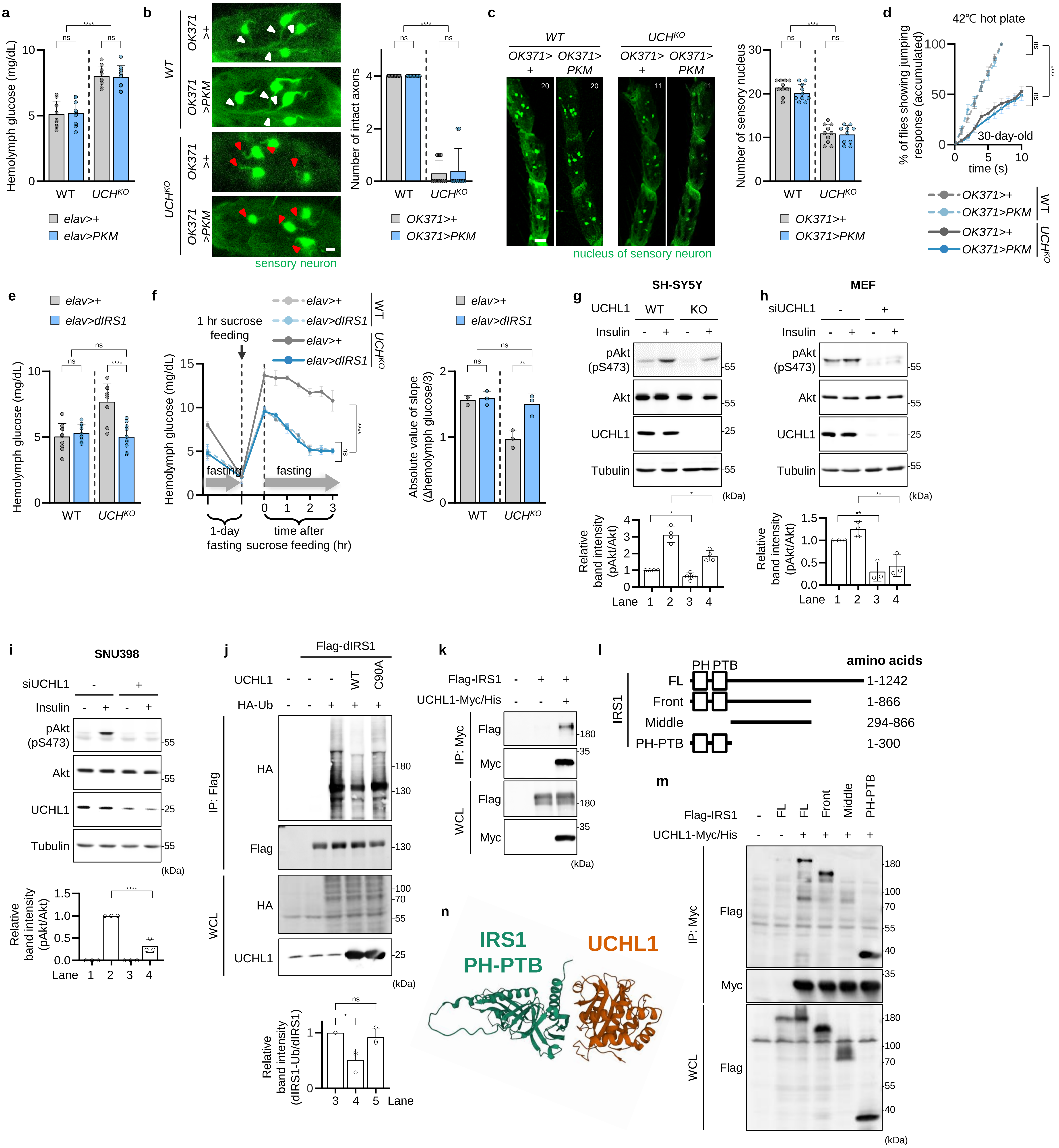
a
Hemolymph glucose (mg/dL)
WT
UCH^KO
elav>+
elav>PKM
b
WT
UCH^KO
OK371 >+
OK371 >PKM
sensory neuron
Number of intact axons
OK371>+
OK371>PKM
c
WT
UCH^KO
OK371> +
OK371> PKM
nucleus of sensory neuron
Number of sensory nucleus
d
42℃ hot plate
% of flies showing jumping response (accumulated)
time (s)
30-day-old
e
elav>+
elav>dIRS1
Hemolymph glucose (mg/dL)
f
1 hr sucrose feeding
fasting
1-day fasting
time after sucrose feeding (hr)
Absolute value of slope (Δhemolymph glucose/3)
g
SH-SY5Y
UCHL1
KO
Insulin
pAkt (pS473)
Akt
Tubulin
(kDa)
Relative band intensity (pAkt/Akt)
Lane
h
MEF
siUCHL1
i
SNU398
j
Flag-dIRS1
HA-Ub
IP: Flag
WCL
HA
Flag
C90A
Relative band intensity (dIRS1-Ub/dIRS1)
k
Flag-IRS1
UCHL1-Myc/His
IP: Myc
Myc
l
PH PTB
amino acids
IRS1
FL
Front
Middle
PH-PTB
1-1242
1-866
294-866
1-300
m
n
IRS1 PH-PTB
UCHL1

Supplementary Fig. 5 | Genetic interaction between UCHL1 and IRS1 in fruit flies and mammalian cells. a, Glucose concentrations in the hemolymph of 3-day-old flies. n = 10. **b,** Left, confocal fluorescence images for axons and somas of sensory neurons of tarsal segments 4 at the front legs of 15-day-old flies expressing *OK371>GFP*. Respective images were obtained from one of the left or right front legs. Green, sensory neuron. White or red triangles indicate intact or impaired axons, respectively. Scale bar, 10 µm. Right, the number of intact axons at tarsal segments 4 at the front legs of 15-day-old flies expressing *OK371>GFP*. n = 10. **c,** Left, confocal fluorescence images of tarsal segments 3, 4, and 5 at the front legs of 30-day-old flies expressing *OK371>nlsGFP*. The number in panels indicates the number of green signals in each image. Respective images were obtained from one of the left or right front legs. Green, the nucleus of sensory neuron. Scale bar, 20 µm. Right, the numbers of green signals at the tarsal segments 3, 4, and 5 of the front legs of 30-day-old flies expressing *OK371>nlsGFP*. n = 10. **d,** Cumulative percentage of 30-day-old flies showing escape responses on the 42℃ hot plate within 10 seconds. n = 100. **e,** Glucose concentrations in the hemolymph of 3-day-old flies. n = 10. **f,** Left, glucose concentrations in the hemolymph of the flies. Each 3-day-old fly was on 10% sucrose solution for 1 hour after 1-day fasting. After the 1-hour sucrose feeding, the hemolymph glucose levels were measured every 30 minutes while fasting the flies again. The indicated statistical significance was calculated from the data after the 1-hour feeding. n = 3. Right, absolute value of the slope of left graph from right after the sucrose feeding to 3 hours after the sucrose feeding (|(hemolymph glucose at 3 hours after sucrose feeding – hemolymph glucose at 0 hours after sucrose feeding)/ 3|). n = 3. **g,** Top, immunoblot analysis of Akt phosphorylation (pS473) in SH-SY5Y *UCHL1* WT and KO cells upon insulin treatment. The cells were treated with 50 nM insulin for 2 hours. Bottom, relative quantification of immunoblot band intensity of anti-pAkt (pS473) normalized to that of anti-Akt. n = 4. **h,** Top, immunoblot analysis of Akt phosphorylation (pS473) in MEF cells transfected with control siRNA or *UCHL1* siRNA (siUCHL1) upon insulin treatment. The cells were treated with 50 nM insulin for 2 hours. Bottom, relative quantification of immunoblot band intensity of anti-pAkt (pS473) normalized to that of anti-Akt. n = 3. **i,** Top, immunoblot analysis of Akt phosphorylation (pS473) in SNU398 cells transfected with control siRNA or *UCHL1* siRNA (siUCHL1) upon insulin treatment. The cells were treated with 50 nM insulin for 2 hours. Bottom, relative quantification of immunoblot band intensity of anti-pAkt (pS473) normalized to that of anti-Akt. n = 3. **j,** Top, immunoblot analysis of dIRS1 ubiquitination in HEK293E cells co-expressing *Drosophila IRS1* (*dIRS1*) and *UCHL1* WT or C90A. The cells were co-transfected with the empty plasmids or the plasmids carrying Flag-tagged *dIRS1*, HA-tagged *Ubiquitin*, and *UCHL1* WT or C90A upon 40 µM MG132 treatment for 4 hours to all samples. Bottom, relative quantification of anti-HA immunoblot band intensity from anti-Flag immunoprecipitation normalized to anti-Flag immunoblot band intensity from anti-Flag immunoprecipitation. n = 3. **k,** Immunoblot analysis of binding between IRS1 and UCHL1 in HEK293E cells co-expressing *IRS1* and *UCHL1*. The cells were co-transfected with the empty plasmids or the plasmids carrying Flag-tagged *IRS1* and Myc/His-tagged *UCHL1*. **l,** Schematic diagram showing truncated forms of IRS1. **m,** Immunoblot analysis of binding between truncated forms of IRS1 and UCHL1 in HEK293T cells co-expressing *IRS1* and *UCHL1*. The cells were co-transfected with the empty plasmids or the plasmids carrying Flag-tagged full

length (FL), PH-PTB, Middle, or Front form of *IRS* and Myc/His-tagged *UCHL1*. **n,** Prediction of the binding between PH-PTB domain of IRS1 and UCHL1 by ColabFold. Green, PH-PTB domain of IRS1. Orange, UCHL1. IP, immunoprecipitation. WCL, whole cell lysate. Data are presented as mean ± SD. Two-way ANOVA with Sidak's multiple comparison test was used (**a**, **b**, **c**, **e**, and **f**). Mantel-Cox test was used (**d**). One-way ANOVA with Holm-Sidak's multiple comparison test was used (**g**). One-way ANOVA with Tukey's multiple comparison test was used (**h** and **j**). Two-tailed paired Student's t-test was used (**i**). *$p < 0.05$. **$p < 0.01$. ****$p < 0.0001$. ns, no significant.

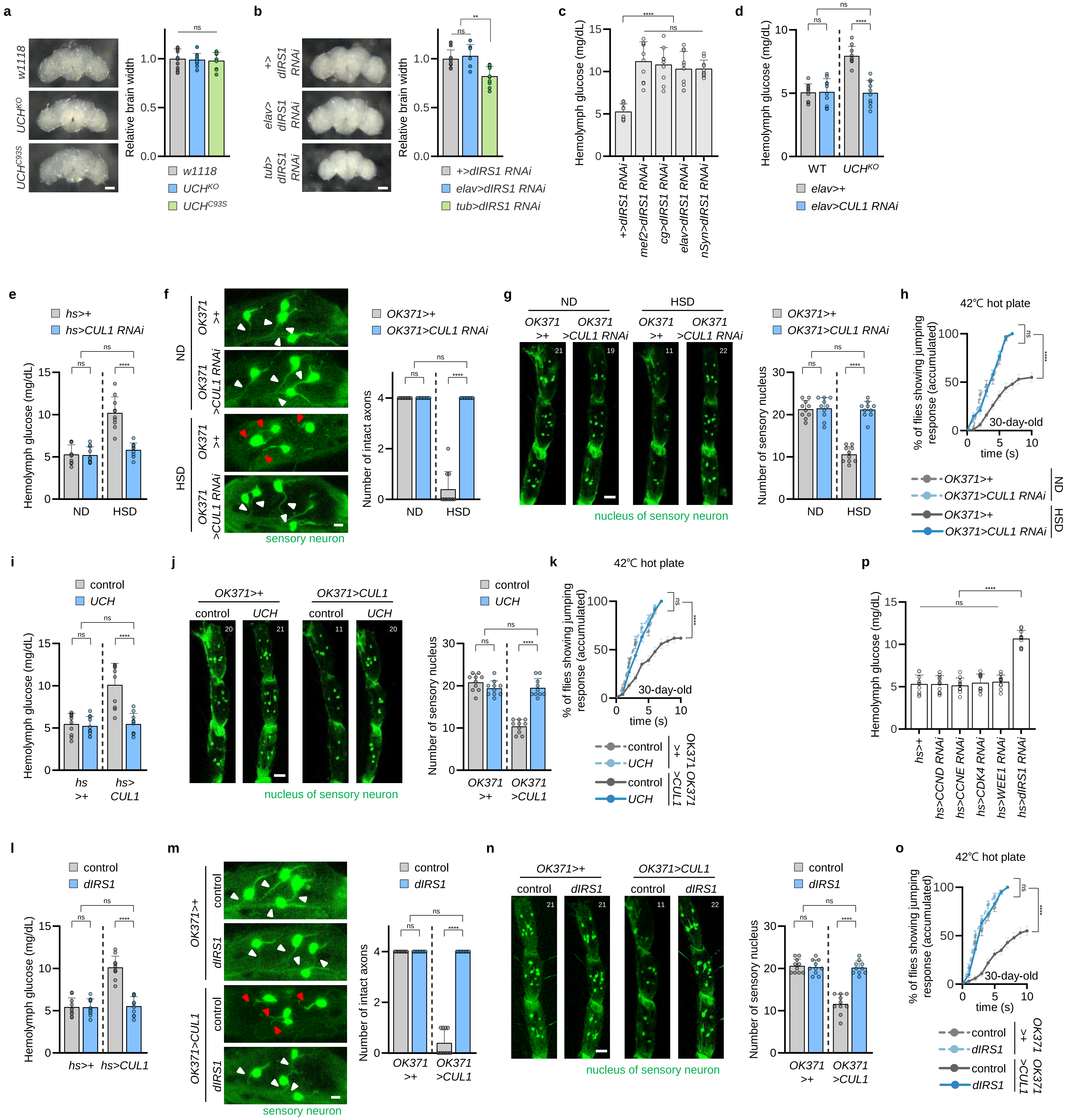
a
w1118
UCH^KO
UCH^C93S
Relative brain width
w1118
UCH^KO
UCH^C93S
b
+> dIRS1 RNAi
elav> dIRS1 RNAi
tub> dIRS1 RNAi
Relative brain width
+>dIRS1 RNAi
elav>dIRS1 RNAi
tub>dIRS1 RNAi
c
Hemolymph glucose (mg/dL)
+>dIRS1 RNAi
mef2>dIRS1 RNAi
cg>dIRS1 RNAi
elav>dIRS1 RNAi
nSyn>dIRS1 RNAi
d
Hemolymph glucose (mg/dL)
WT
UCH^KO
elav>+
elav>CUL1 RNAi
e
hs>+
hs>CUL1 RNAi
Hemolymph glucose (mg/dL)
ND
HSD
f
ND
HSD
OK371 >+
OK371 >CUL1 RNAi
sensory neuron
OK371>+
OK371>CUL1 RNAi
Number of intact axons
g
ND
HSD
OK371 >+
OK371 >CUL1 RNAi
nucleus of sensory neuron
OK371>+
OK371>CUL1 RNAi
Number of sensory nucleus
h
42℃ hot plate
% of flies showing jumping response (accumulated)
30-day-old
time (s)
OK371>+
OK371>CUL1 RNAi
i
control
UCH
Hemolymph glucose (mg/dL)
hs >+
hs> CUL1
j
OK371>+
OK371>CUL1
control
UCH
nucleus of sensory neuron
Number of sensory nucleus
OK371 >+
OK371 >CUL1
k
42℃ hot plate
% of flies showing jumping response (accumulated)
30-day-old
time (s)
control
UCH
p
Hemolymph glucose (mg/dL)
hs>+
hs>CCND RNAi
hs>CCNE RNAi
hs>CDK4 RNAi
hs>WEE1 RNAi
hs>dIRS1 RNAi
l
control
dIRS1
Hemolymph glucose (mg/dL)
hs>+
hs>CUL1
m
OK371>+
OK371>CUL1
control
dIRS1
sensory neuron
Number of intact axons
n
OK371>+
OK371>CUL1
control
dIRS1
nucleus of sensory neuron
Number of sensory nucleus
o
42℃ hot plate
% of flies showing jumping response (accumulated)
30-day-old
time (s)
OK371 >+
OK371 >CUL1

Supplementary Fig. 6 | Genetic interactions between *UCHL1* and *CUL1* in fruit flies. a, Left, images of the fly brain with indicated genotypes. Right, relative width of brain. Normalized to the brain width of *w1118*. n = 10. Scale bar, 0.1 mm. **b,** Left, images of the fly brain with indicated genotypes. Right, relative width of brain. Normalized to the brain width of *+>dIRS1 RNAi*. n = 10. Scale bar, 0.1 mm. **c,** Glucose concentrations in the hemolymph of 3-day-old flies. n = 10. **d,** Glucose concentrations in the hemolymph of 3-day-old flies. n = 10. **e,** Glucose concentrations in the hemolymph of 3-day-old flies upon ND or HSD. n = 10. **f,** Left, confocal fluorescence images for axons and somas of sensory neurons of tarsal segments 4 at the front legs of 15-day-old flies expressing *OK371>GFP*. Respective images were obtained from one of the left or right front legs. Green, sensory neuron. White or red triangles indicate intact or impaired axons, respectively. Scale bar, 10 μm. Right, the number of intact axons at tarsal segments 4 at the front legs of 15-day-old flies expressing *OK371>GFP*. n = 10. **g,** Left, confocal fluorescence images of tarsal segments 3, 4, and 5 at the front legs of 30-day-old flies expressing *OK371>nlsGFP* upon ND or HSD. The number in panels indicates the number of green signals in each image. Respective images were obtained from one of the left or right front legs. Green, the nucleus of sensory neuron. Scale bar, 20 μm. Right, the numbers of green signals at the tarsal segments 3, 4, and 5 of the front legs of 30-day-old flies expressing *OK371>nlsGFP* upon ND or HSD. n = 10. **h,** Cumulative percentage of 30-day-old flies upon ND or HSD showing escape responses on the 42°C hot plates within 10 seconds. n = 100. **i,** Glucose concentrations in the hemolymph of 3-day-old flies. n = 10. **j,** Left, confocal fluorescence images of tarsal segments 3, 4, and 5 at the front legs of 30-day-old flies expressing *OK371>nlsGFP*. The number in panels indicates the number of green signals in each image. Respective images were obtained from one of the left or right front legs. Green, the nucleus of sensory neuron. Scale bar, 20 μm. Right, the numbers of green signals at the tarsal segments 3, 4, and 5 of the front legs of 30-day-old flies expressing *OK371>nlsGFP*. n = 10. **k,** Cumulative percentage of 30-day-old flies showing escape responses on the 42°C hot plates within 10 seconds. n = 100. **l,** Glucose concentrations in the hemolymph of 3-day-old flies. n = 10. **m,** Left, confocal fluorescence images for axons and somas of sensory neurons of tarsal segments 4 at the front legs of 15-day-old flies expressing *OK371>GFP*. Respective images were obtained from one of the left or right front legs. Green, sensory neuron. White or red triangles indicate intact or impaired axons, respectively. Scale bar, 10 μm. Right, the number of intact axons at tarsal segments 4 at the front legs of 15-day-old flies expressing *OK371>GFP*. n = 10. **n,** Left, confocal fluorescence images of tarsal segments 3, 4, and 5 at the front legs of 30-day-old flies expressing *OK371>nlsGFP*. The number in panels indicates the number of green signals in each image. Respective images were obtained from one of the left or right front legs. Green, the nucleus of sensory neuron. Scale bar, 20 μm. Right, the numbers of green signals at the tarsal segments 3, 4, and 5 of the front legs of 30-day-old flies expressing *OK371>nlsGFP*. n = 10. **o,** Cumulative percentage of 30-day-old flies showing escape responses on the 42°C hot plates within 10 seconds. n = 100. **p,** Glucose concentrations in the hemolymph of 3-day-old flies. n = 10. ND, normal diet. HSD, high-sucrose diet. Data are presented as mean ± SD. One-way ANOVA with Tukey's multiple comparison test was used (**a**, **b**, **c**, and **p**). Two-way ANOVA with Sidak's multiple comparison test was used (**d**, **e**, **f**, **g**, **i**, **j**, **l**, **m** and **n**). Mantel-Cox test was used (**h, k,** and **o**). **p < 0.01. ****p < 0.0001. ns, no significant.

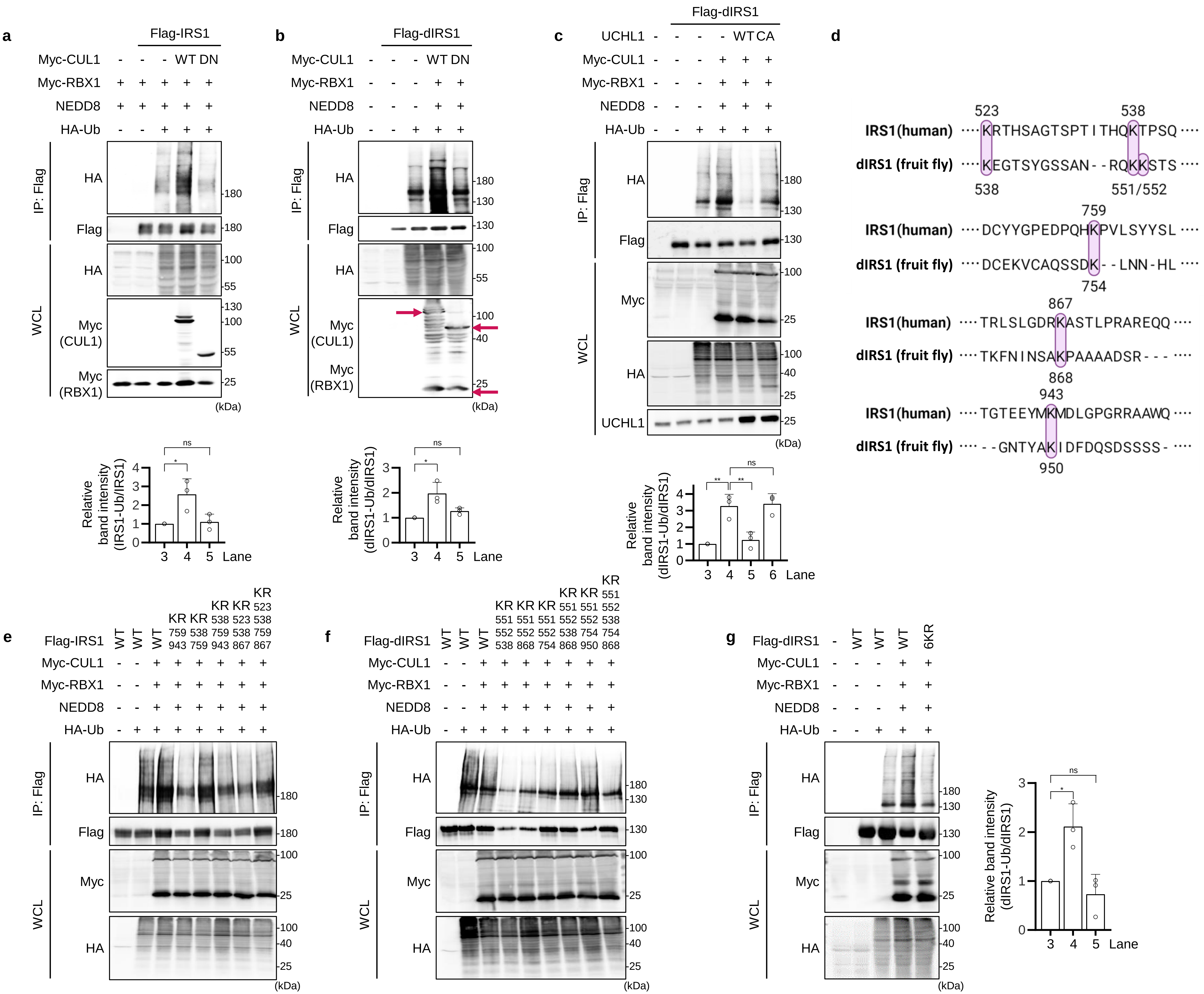
a
Flag-IRS1
Myc-CUL1
Myc-RBX1
NEDD8
HA-Ub
WT DN
IP: Flag
WCL
HA
Flag
Myc (CUL1)
Myc (RBX1)
(kDa)
Relative band intensity (IRS1-Ub/IRS1)
Lane
b
Flag-dIRS1
Relative band intensity (dIRS1-Ub/dIRS1)
c
UCHL1
WT CA
d
IRS1(human)
dIRS1 (fruit fly)
523
538
551/552
759
754
867
868
943
950
····KRTHSAGTSPTITHQKTPSQ····
····KEGTSYGSSAN--RQKKSTS····
····DCYYGPEDPQHKPVLSYYSL····
····DCEKVCAQSSDK--LNN-HL····
····TRLSLGDRKASTLPRAREQQ····
····TKFNINSAKPAAAADSR---····
····TGTEEYMKMDLGPGRRAAWQ····
····--GNTYAKIDFDQSDSSSS-····
e
f
g
6KR
ns

Supplementary Fig. 7 | CUL1 ubiquitinates IRS1, which is antagonized by UCHL1. a, Top, immunoblot analysis of IRS1 ubiquitination in HEK293E cells co-expressing *IRS1*, *CUL1*, *RBX1*, and *NEDD8*. The cells were co-transfected with the empty plasmids or the plasmids carrying Flag-tagged *IRS1*, Myc-tagged *CUL1*, Myc-tagged *RBX1*, and *NEDD8*, and HA-tagged *Ubiquitin* (*Ub*) upon 40 μM MG132 treatment for 4 hours to all samples. Bottom, relative quantification of anti-HA immunoblot band intensity from anti-Flag immunoprecipitation normalized to anti-Flag immunoblot band intensity from anti-Flag immunoprecipitation. n = 3. **b,** Top, immunoblot analysis of dIRS1 ubiquitination in HEK293E cells co-expressing *dIRS1, CUL1*, *RBX1*, and *NEDD8*. The cells were co-transfected with the empty plasmids or the plasmids carrying Flag-tagged *dIRS1*, Myc-tagged *CUL1* WT or DN, Myc-tagged *RBX1*, *NEDD8*, and HA-tagged *Ubiquitin* upon 40 μM MG132 treatment for 4 hours to all samples. Bottom, relative quantification of anti-HA immunoblot band intensity from anti-Flag immunoprecipitation normalized to anti-Flag immunoblot band intensity from anti-Flag immunoprecipitation. n = 3. **c,** Top, immunoblot analysis of dIRS1 ubiquitination in HEK293E cells co-expressing *dIRS1*, *UCHL1*, *CUL1*, *RBX1*, and *NEDD8*. The cells were co-transfected with the empty plasmids or the plasmids carrying Flag-tagged *dIRS1*, Myc-tagged *CUL1*, Myc-tagged *RBX1*, and *NEDD8*, HA-tagged *Ubiquitin*, and *UCHL1* WT or C90A upon 40 μM MG132 treatment to all samples. Bottom, relative quantification of anti-HA immunoblot band intensity from anti-Flag immunoprecipitation normalized to anti-Flag immunoblot band intensity from anti-Flag immunoprecipitation. n = 3. **d,** Alignment of the protein sequences between human IRS1 and *Drosophila* IRS1 (dIRS1). The illustration was created using Biorender.com. **e,** Immunoblot analysis of IRS1 ubiquitination in HEK293E cells co-expressing various KR mutant forms of *IRS1*, *CUL1*, *RBX1*, and *NEDD8*. The cells were co-transfected with the empty plasmids or the plasmids carrying Flag-tagged *IRS1*, Myc-tagged *CUL1*, Myc-tagged *RBX1*, *NEDD8*, and HA-tagged *Ubiquitin* (Ub) upon 40 μM MG132 treatment for 4 hours to all samples. **f,** Immunoblot analysis of dIRS1 ubiquitination in HEK293E cells co-expressing various KR mutant forms of *dIRS1*, *CUL1*, *RBX1*, and *NEDD8*. The cells were co-transfected with the empty plasmids or the plasmids carrying Flag-tagged *dIRS1*, Myc-tagged *CUL1*, Myc-tagged *RBX1*, *NEDD8*, and HA-tagged *Ubiquitin* (Ub) upon 40 μM MG132 treatment for 4 hours to all samples. **g,** Left, immunoblot analysis of dIRS1 ubiquitination in HEK293E cells co-expressing *dIRS1* WT or 6KR (K538, 551/552, 754, 868, and 950R), *CUL1*, *RBX1*, and *NEDD8*. The cells were co-transfected with the empty plasmids or the plasmids carrying Flag-tagged *dIRS1* WT or 6KR, Myc-tagged *CUL1*, Myc-tagged *RBX1*, *NEDD8*, and HA-tagged *Ubiquitin* (Ub) upon 40 μM MG132 treatment for 4 hours to all samples. Right, relative quantification of anti-HA immunoblot band intensity from anti-Flag immunoprecipitation normalized to anti-Flag immunoblot band intensity from anti-Flag immunoprecipitation. n = 3. IP, immunoprecipitation. WCL, whole cell lysate. Data are presented as mean ± SD. One-way ANOVA with Tukey's multiple comparison test was used (**a**, **b**, **c**, and **g**). $^{*}p < 0.05$, $^{**}p < 0.01$. ns, no significant.

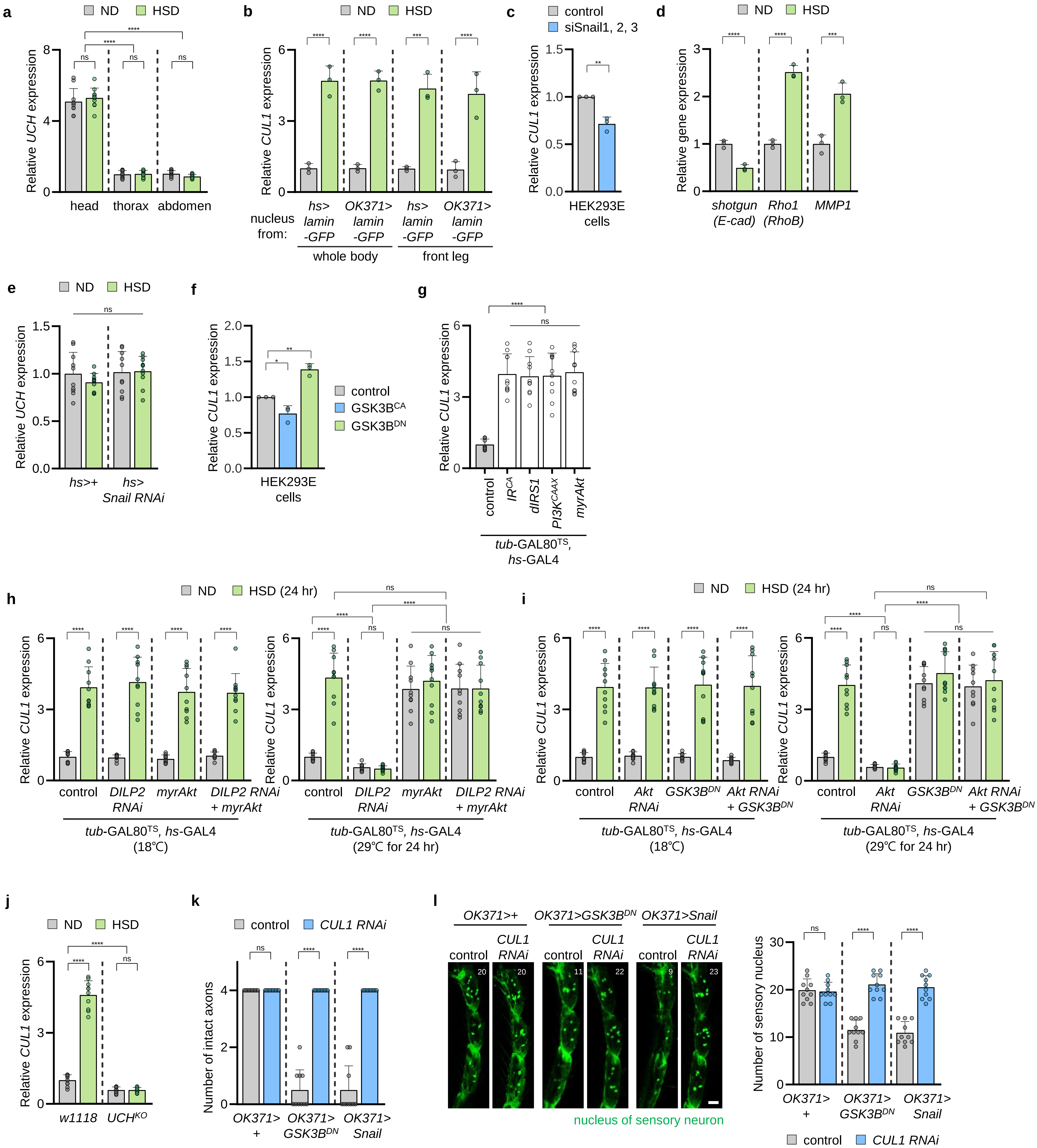
a
ND HSD
Relative UCH expression
head thorax abdomen
b
ND HSD
Relative CUL1 expression
nucleus from:
hs> lamin -GFP
OK371> lamin -GFP
whole body
front leg
c
control
siSnail1, 2, 3
Relative CUL1 expression
HEK293E cells
d
ND HSD
Relative gene expression
shotgun (E-cad)
Rho1 (RhoB)
MMP1
e
ND HSD
Relative UCH expression
hs>+
hs> Snail RNAi
f
Relative CUL1 expression
control
GSK3B^{CA}
GSK3B^{DN}
HEK293E cells
g
Relative CUL1 expression
control
IR^{CA}
dIRS1
PI3K^{CAAX}
myrAkt
tub-GAL80^{TS}, hs-GAL4
h
ND HSD (24 hr)
Relative CUL1 expression
control
DILP2 RNAi
myrAkt
DILP2 RNAi + myrAkt
tub-GAL80^{TS}, hs-GAL4 (18℃)
tub-GAL80^{TS}, hs-GAL4 (29℃ for 24 hr)
i
ND HSD (24 hr)
Relative CUL1 expression
control
Akt RNAi
GSK3B^{DN}
Akt RNAi + GSK3B^{DN}
tub-GAL80^{TS}, hs-GAL4 (18℃)
tub-GAL80^{TS}, hs-GAL4 (29℃ for 24 hr)
j
ND HSD
Relative CUL1 expression
w1118
UCH^{KO}
k
control CUL1 RNAi
Number of intact axons
OK371> +
OK371> GSK3B^{DN}
OK371> Snail
l
OK371>+
OK371>GSK3B^{DN}
OK371>Snail
control
CUL1 RNAi
20 20 11 22 9 23
nucleus of sensory neuron
Number of sensory nucleus
OK371> +
OK371> GSK3B^{DN}
OK371> Snail
control CUL1 RNAi

Supplementary Fig. 8 | GSK3B and Snail regulate *CUL1* transcription in response to HSD. **a,** Relative expressions of *UCH* normalized to *rp49* expression from the head, thorax, and abdomen of *w1118* upon ND or HSD. n = 10. **b,** Relative expressions of *CUL1* normalized to *rp49* expression from extracted GFP^{+} nuclei of whole bodies or front legs of the flies upon ND or HSD. n = 3. **c,** Relative expressions of *CUL1* normalized to *rp30* expression from HEK293E cells. n = 3. **d,** Relative expressions of *shotgun*, *Rho1*, and *MMP1* normalized to *rp49* expression from the flies upon ND or HSD. n = 3. **e,** Relative expressions of *UCH* normalized to *rp49* expression from the flies upon ND or HSD. n = 10. **f,** Relative expressions of *CUL1* normalized to *rp30* expression from HEK293E cells. n = 3. **g,** Relative expressions of *CUL1* normalized to *rp49* expression from the flies. Exogenous expression of InR^{CA}, *dIRS1*, $PI3K^{CAAX}$, or *myrAkt* was induced for 12 hours by placing the flies at 29°C. RNA extractions were performed right after the induction. n = 10. **h,** Relative expressions of *CUL1* normalized to *rp49* expressions from the flies upon ND or HSD. n = 10. Flies of indicated genotypes were raised at 18°C. Afterward, the flies were placed at 29°C to induce exogenous expression of *DILP*2 *RNAi*, *myrAkt*, or both constructs for 24 hours. During this time, HSD groups of flies were fed a high sucrose-containing diet for 24 hours. RNA extractions were then performed right after the 24-hour induction and HSD. n = 10. **i,** Relative expressions of *CUL1* normalized to *rp49* expressions from the flies upon ND or HSD. n = 10. Flies of indicated genotypes were raised at 18°C. Afterward, the flies were placed at 29°C to induce exogenous expression of *Akt RNAi*, $GSK3B^{DN}$, or both constructs for 24 hours. During this time, HSD groups of flies were fed a high sucrose-containing diet for 24 hours. RNA extractions were then performed right after the 24-hour induction and HSD. n = 10. **j,** Relative expressions of *CUL1* normalized to *rp49* expression from the head of the flies upon ND or HSD. n = 10. **k,** The number of intact axons at tarsal segments 4 at the front legs of 15-day-old flies expressing *OK371>GFP*. Quantification data of Fig. 6g. n = 10. **l,** Left, confocal fluorescence images of tarsal segments 3, 4, and 5 at the front legs of 30-day-old flies expressing *OK371>nlsGFP*. The number in panels indicates the number of green signals in each image. Respective images were obtained from one of the left or right front legs. Green, the nucleus of sensory neuron. Scale bar, 20 μm. Right, the numbers of green signals at the tarsal segments 3, 4, and 5 of the front legs of 30-day-old flies expressing *OK371>nlsGFP*. n = 10. ND, normal diet. HSD, high-sucrose diet. Data are presented as mean ± SD. Two-way ANOVA with Sidak's multiple comparison test was used (**a**, **e**, **h**, **i**, **j**, **k**, and **l**). One-way ANOVA with Tukey's multiple comparison test was used (**b**, **d**, **f**, and **g**). Two-tailed paired Student's t-test was used (**c**). *p < 0.05. **p < 0.01. ***p < 0.001. ****p < 0.0001. ns, no significant.

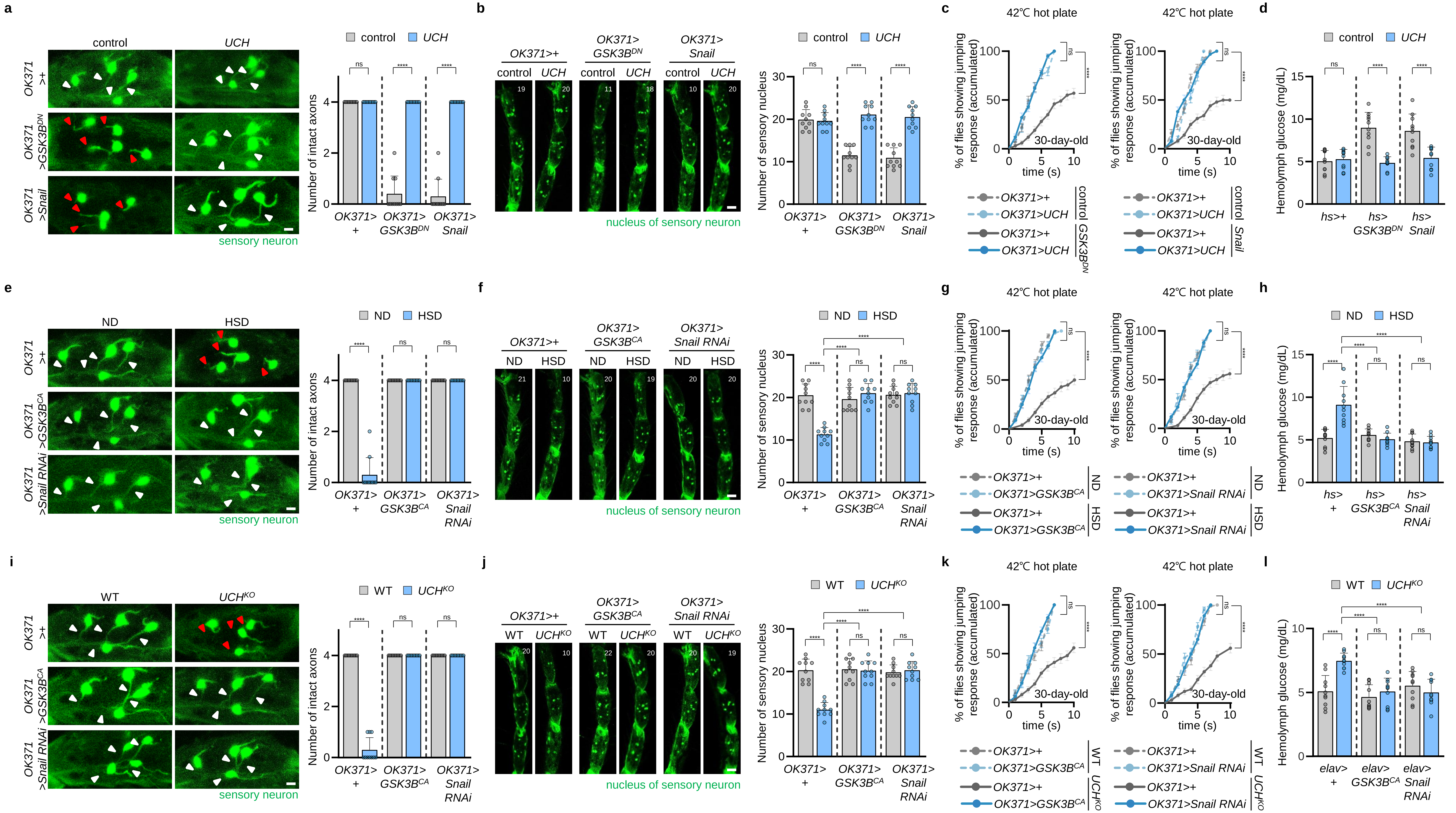
a
control
UCH
OK371 >+
OK371 >GSK3B^DN
OK371 >Snail
sensory neuron
Number of intact axons
OK371> +
OK371> GSK3B^DN
OK371> Snail
b
OK371>+
OK371> GSK3B^DN
OK371> Snail
nucleus of sensory neuron
Number of sensory nucleus
c
42℃ hot plate
% of flies showing jumping response (accumulated)
time (s)
30-day-old
OK371>+
OK371>UCH
control
GSK3B^DN
Snail
d
Hemolymph glucose (mg/dL)
hs>+
hs> GSK3B^DN
hs> Snail
e
ND
HSD
OK371 >+
OK371 >GSK3B^CA
OK371 >Snail RNAi
sensory neuron
Number of intact axons
OK371> GSK3B^CA
OK371> Snail RNAi
f
nucleus of sensory neuron
Number of sensory nucleus
g
42℃ hot plate
30-day-old
OK371>GSK3B^CA
OK371>Snail RNAi
h
Hemolymph glucose (mg/dL)
hs> +
hs> GSK3B^CA
hs> Snail RNAi
i
WT
UCH^KO
sensory neuron
j
nucleus of sensory neuron
k
42℃ hot plate
30-day-old
l
elav> +
elav> GSK3B^CA
elav> Snail RNAi

Supplementary Fig. 9 | Genetic interaction between *UCHL1* and *GSK3B* or *Snail* in fruit flies. a, Left, confocal fluorescence images for axons and somas of sensory neurons of tarsal segments 4 at the front legs of 15-day-old flies expressing *OK371>GFP*. Respective images were obtained from one of the left or right front legs. Green, sensory neuron. White or red triangles indicate intact or impaired axons, respectively. Scale bar, 10 μm. Right, the number of intact axons at tarsal segments 4 at the front legs of 15-day-old flies expressing *OK371>GFP*. n = 10. **b,** Left, confocal fluorescence images of tarsal segments 3, 4, and 5 at the front legs of 30-day-old flies expressing *OK371>nlsGFP*. The number in panels indicates the number of green signals in each image. Respective images were obtained from one of the left or right front legs. Green, the nucleus of sensory neuron. Scale bar, 20 μm. Right, the numbers of green signals at the tarsal segments 3, 4, and 5 of the front legs of 30-day-old flies expressing *OK371>nlsGFP*. n = 10. **c,** Cumulative percentage of 30-day-old flies showing escape responses on the 42°C hot plates within 10 seconds. n = 100. **d,** Glucose concentrations in the hemolymph of 3-day-old flies. n = 10. **e,** Left, confocal fluorescence images for axons and somas of sensory neurons of tarsal segments 4 at the front legs of 15-day-old flies expressing *OK371>GFP* upon ND or HSD. Respective images were obtained from one of the left or right front legs. Green, sensory neuron. White or red triangles indicate intact or impaired axons, respectively. Scale bar, 10 μm. Right, the number of intact axons at tarsal segments 4 at the front legs of 15-day-old flies expressing *OK371>GFP* upon ND or HSD. n = 10. **f,** Left, confocal fluorescence images of tarsal segments 3, 4, and 5 at the front legs of 30-day-old flies expressing *OK371>nlsGFP* upon ND or HSD. The number in panels indicates the number of green signals in each image. Respective images were obtained from one of the left or right front legs. Green, the nucleus of sensory neuron. Scale bar, 20 μm. Right, the numbers of green signals at the tarsal segments 3, 4, and 5 of the front legs of 30-day-old flies expressing *OK371>nlsGFP* upon ND or HSD. n = 10. **g,** Cumulative percentage of 30-day-old flies upon ND or HSD showing escape responses on the 42°C hot plates within 10 seconds. n = 100. **h,** Glucose concentrations in the hemolymph of 3-day-old flies upon ND or HSD. n = 10. **i,** Left, confocal fluorescence images for axons and somas of sensory neurons of tarsal segments 4 at the front legs of 15-day-old flies expressing *OK371>GFP*. Respective images were obtained from one of the left or right front legs. Green, sensory neuron. White or red triangles indicate intact or impaired axons, respectively. Scale bar, 10 μm. Right, the number of intact axons at tarsal segments 4 at the front legs of 15-day-old flies expressing *OK371>GFP*. n = 10. **j,** Left, confocal fluorescence images of tarsal segments 3, 4, and 5 at the front legs of 30-day-old flies expressing *OK371>nlsGFP*. The number in panels indicates the number of green signals in each image. Respective images were obtained from one of the left or right front legs. Green, the nucleus of sensory neuron. Scale bar, 20 μm. Right, the numbers of green signals at the tarsal segments 3, 4, and 5 of the front legs of 30-day-old flies expressing *OK371>nlsGFP*. n = 10. **k,** Cumulative percentage of 30-day-old flies showing escape responses on the 42°C hot plates within 10 seconds. n = 100. **l,** Glucose concentrations in the hemolymph of 3-day-old flies. n = 10. ND, normal diet. HSD, high-sucrose diet. Data are presented as mean ± SD. Two-way ANOVA with Sidak's multiple comparison test was used (**a**, **b**, **d**, **e**, **f**, **h**, **i**, **j**, and **l**). Mantel-Cox test was used (**c**, **g**, and **k**). ****p < 0.0001. ns, no significant.

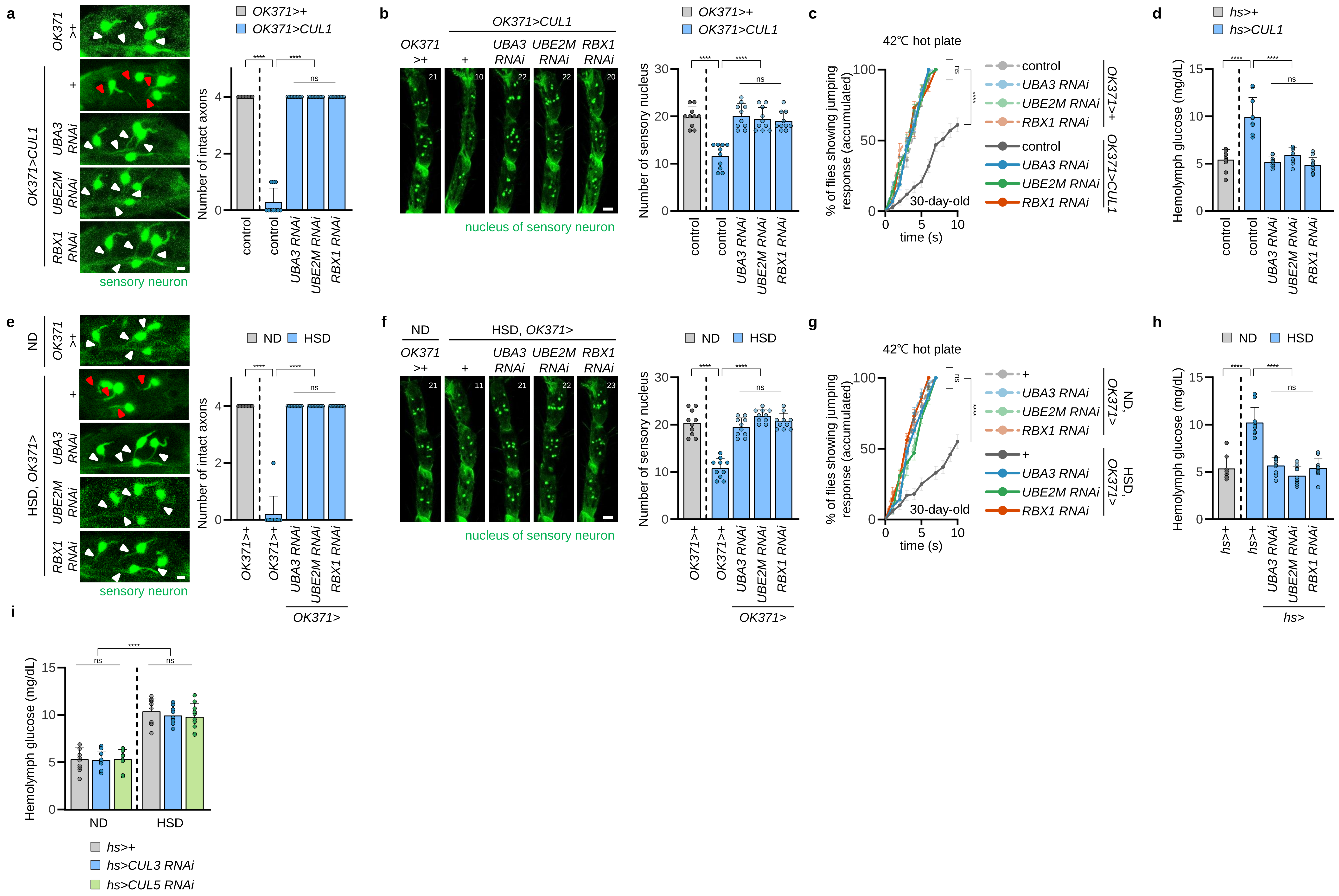

a
OK371>+
OK371>CUL1
OK371>+
OK371>CUL1
Number of intact axons
control
UBA3 RNAi
UBE2M RNAi
RBX1 RNAi
sensory neuron
b
OK371>CUL1
nucleus of sensory neuron
Number of sensory nucleus
c
42℃ hot plate
% of flies showing jumping response (accumulated)
time (s)
30-day-old
d
hs>+
hs>CUL1
Hemolymph glucose (mg/dL)
e
ND
HSD
HSD, OK371>
f
HSD, OK371>
g
h
i
hs>CUL3 RNAi
hs>CUL5 RNAi

Supplementary Fig. 10 | Genetic inhibition of neddylation enzymes rescues T2D- or DSN-related defects in fruit flies. a, Left, confocal fluorescence images for axons and somas of sensory neurons of tarsal segments 4 at the front legs of 15-day-old flies expressing *OK371>GFP*. Respective images were obtained from one of the left or right front legs. Green, sensory neuron. White or red triangles indicate intact or impaired axons, respectively. Scale bar, 10 μm. Right, the number of intact axons at tarsal segments 4 at the front legs of 15-day-old flies expressing *OK371>GFP*. n = 10. **b,** Left, confocal fluorescence images of tarsal segments 3, 4, and 5 at the front legs of 30-day-old flies expressing *OK371>nlsGFP*. The number in panels indicates the number of green signals in each image. Respective images were obtained from one of the left or right front legs. Green, the nucleus of sensory neuron. Scale bar, 20 μm. Right, the numbers of green signals at the tarsal segments 3, 4, and 5 of the front legs of 30-day-old flies expressing *OK371>nlsGFP*. n = 10. **c,** Cumulative percentage of 30-day-old flies showing escape responses on the 42°C hot plates within 10 seconds. n = 100. **d,** Glucose concentrations in the hemolymph of 3-day-old flies. n = 10. **e,** Left, confocal fluorescence images for axons and somas of sensory neurons of tarsal segments 4 at the front legs of 15-day-old flies expressing *OK371>GFP* upon ND or HSD. Respective images were obtained from one of the left or right front legs. Green, sensory neuron. White or red triangles indicate intact or impaired axons, respectively. Scale bar, 10 μm. Right, the number of intact axons at tarsal segments 4 at the front legs of 15-day-old flies expressing *OK371>GFP* upon ND or HSD. n = 10. **f,** Left, confocal fluorescence images of tarsal segments 3, 4, and 5 at the front legs of 30-day-old flies expressing *OK371>nlsGFP* upon ND or HSD. The number in panels indicates the number of green signals in each image. Respective images were obtained from one of the left or right front legs. Green, the nucleus of sensory neurons. Scale bar, 20 μm. Right, the numbers of green signals at the tarsal segments 3, 4, and 5 of the front legs of 30-day-old flies expressing *OK371>nlsGFP* upon ND or HSD. n = 10. **g,** Cumulative percentage of 30-day-old flies upon ND or HSD showing escape responses on the 42°C hot plates within 10 seconds. n = 100. **h,** Glucose concentrations in the hemolymph of 3-day-old flies upon ND or HSD. n = 10. **i,** Glucose concentrations in the hemolymph of 3-day-old flies upon ND or HSD. n = 10. ND, normal diet. HSD, high-sucrose diet. Data are presented as mean ± SD. One-way ANOVA with Tukey's multiple comparison test was used (**a**, **b**, **d**, **e**, **f**, and **h**). Mantel-Cox test was used (**c** and **g**). Two-way ANOVA with Sidak's multiple comparison test was used (**i**). ****$p < 0.0001$. ns, no significant.

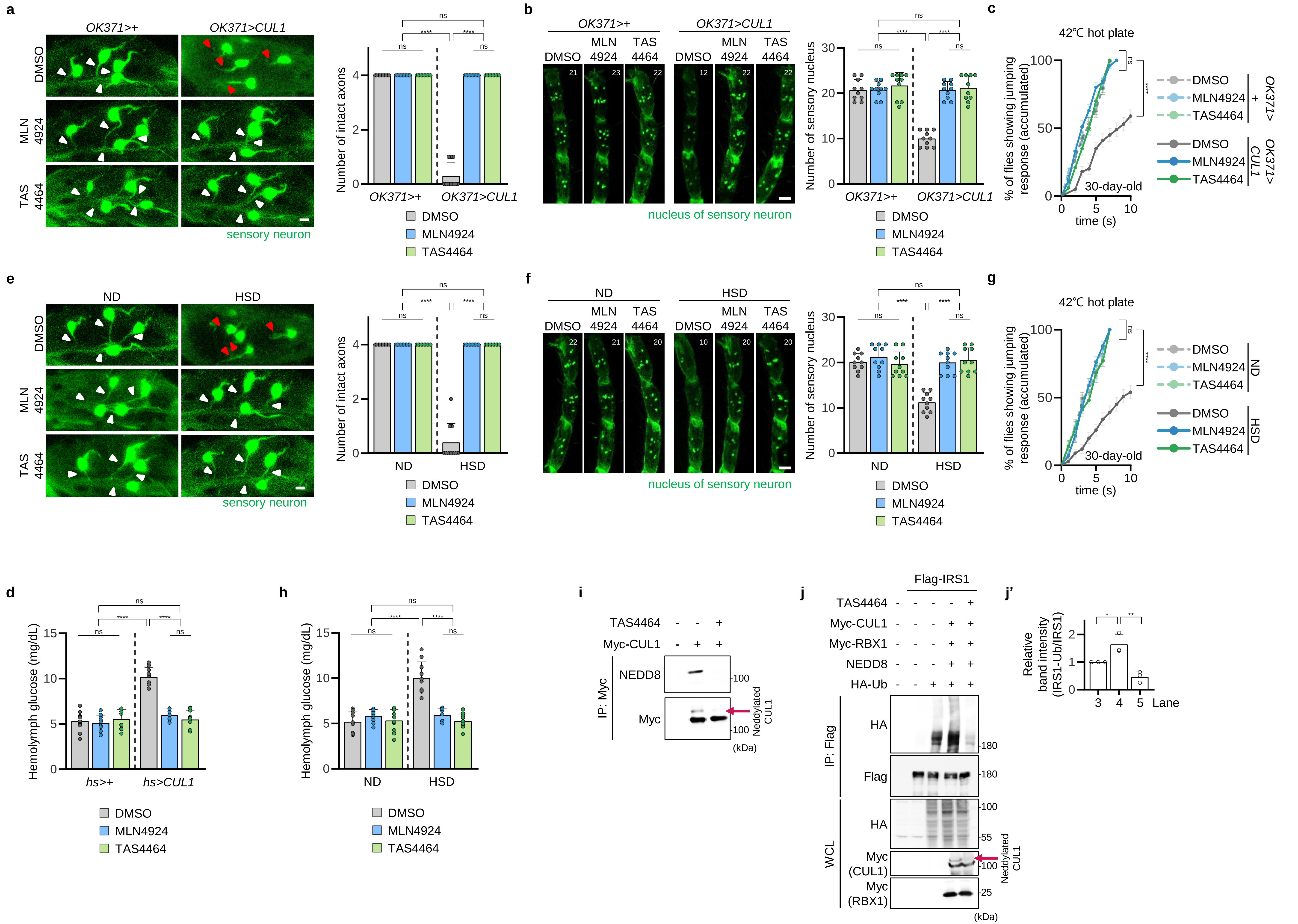

a
OK371>+
OK371>CUL1
DMSO
MLN 4924
TAS 4464
sensory neuron
Number of intact axons
OK371>+
OK371>CUL1
DMSO
MLN4924
TAS4464
b
OK371>+
OK371>CUL1
DMSO
MLN 4924
TAS 4464
nucleus of sensory neuron
Number of sensory nucleus
c
42℃ hot plate
% of flies showing jumping response (accumulated)
time (s)
30-day-old
DMSO
MLN4924
TAS4464
OK371>+
OK371>CUL1
e
ND
HSD
sensory neuron
f
nucleus of sensory neuron
g
d
Hemolymph glucose (mg/dL)
hs>+
hs>CUL1
h
i
TAS4464
Myc-CUL1
IP: Myc
NEDD8
Myc
Neddylated CUL1
(kDa)
j
Flag-IRS1
TAS4464
Myc-CUL1
Myc-RBX1
NEDD8
HA-Ub
IP: Flag
HA
Flag
WCL
Myc (CUL1)
Myc (RBX1)
j'
Relative band intensity (IRS1-Ub/IRS1)
Lane

Supplementary Fig. 11 | Administration of MLN4924 or TAS4464 rescues T2D- or DN-like phenotypes. a, Left, confocal fluorescence images for axons and somas of sensory neurons of tarsal segments 4 at the front legs of 15-day-old flies expressing *OK371>GFP*. The flies were fed 0.05% DMSO, 20 μM MLN4924, or 20 μM TAS4464 continuously after eclosion. Respective images were obtained from one of the left or right front legs. Green, sensory neuron. White or red triangles indicate intact or impaired axons, respectively. Scale bar, 10 μm. Right, the number of intact axons at tarsal segments 4 at the front legs of 15-day-old flies expressing *OK371>GFP*. n = 10. **b,** Left, confocal fluorescence images of tarsal segments 3, 4, and 5 at the front legs of 30-day-old flies expressing *OK371>nlsGFP*. The flies were fed 0.05% DMSO, 20 μM MLN4924, or 20 μM TAS4464 continuously after eclosion. The number in panels indicates the number of green signals in each image. Respective images were obtained from one of the left or right front legs. Green, the nucleus of sensory neuron. Scale bar, 20 μm. Right, the numbers of green signals at the tarsal segments 3, 4, and 5 of the front legs of 30-day-old flies expressing *OK371>nlsGFP*. n = 10. **c,** Cumulative percentage of 30-day-old flies showing escape responses on the 42°C hot plates within 10 seconds. The flies were fed 0.05% DMSO, 20 μM MLN4924, or 20 μM TAS4464 continuously after eclosion. n = 100. **d,** Glucose concentrations in the hemolymph of 3-day-old flies. The flies were fed 0.05% DMSO, 20 μM MLN4924, or 20 μM TAS4464 continuously after eclosion. n = 10. **e,** Left, confocal fluorescence images for axons and somas of sensory neurons of tarsal segments 4 at the front legs of 15-day-old flies expressing *OK371>GFP* upon ND or HSD. The flies were fed 0.05% DMSO, 20 μM MLN4924, or 20 μM TAS4464 continuously after eclosion. Respective images were obtained from one of the left or right front legs. Green, sensory neuron. White or red triangles indicate intact or impaired axons, respectively. Scale bar, 10 μm. Right, the number of intact axons at tarsal segments 4 at the front legs of 15-day-old flies expressing *OK371>GFP* upon ND or HSD. n = 10. **f,** Left, confocal fluorescence images of tarsal segments 3, 4, and 5 at the front legs of 30-day-old flies expressing *OK371>nlsGFP* upon ND or HSD. The flies were fed the food containing high sucrose and 0.05% DMSO, 20 μM MLN4924, or 20 μM TAS4464 continuously after eclosion. The number in panels indicates the number of green signals in each image. Respective images were obtained from one of the left or right front legs. Green, the nucleus of sensory neurons. Scale bar, 20 μm. Right, the numbers of green signals at the tarsal segments 3, 4, and 5 of the front legs of 30-day-old flies expressing *OK371>nlsGFP* upon ND or HSD. n = 10. **g,** Cumulative percentage of 30-day-old flies upon ND or HSD showing escape responses on the 42°C hot plates within 10 seconds. The flies were fed the food containing high sucrose and 0.05% DMSO, 20 μM MLN4924, or 20 μM TAS4464 continuously after eclosion. n = 100. **h,** Glucose concentrations in the hemolymph of 3-day-old flies upon ND or HSD. The flies were fed 0.05% DMSO, 20 μM MLN4924, or 20 μM TAS4464 continuously after eclosion. n = 10. **i,** Immunoblot analysis of CUL1 neddylation in HEK293E cells. The cells transfected with the empty plasmids or the plasmids carrying Myc-tagged *CUL1* were treated with 10 μM TAS4464 for 4 hours. **j,** Immunoblot analysis of IRS1 ubiquitination in HEK293E cells expressing *CUL1*, *RBX1*, and *NEDD8*. The cells were co-transfected with the empty plasmids or the plasmids carrying Flag-tagged *IRS1*, Myc-tagged *CUL1*, Myc-tagged *RBX1*, *NEDD8*, and HA-tagged *Ubiquitin* upon 40 μM MG132 for 4 hours to all samples and 10 μM TAS4464 treatment for 4 hours. **j'**, Relative quantification of anti-HA immunoblot band intensity from anti-Flag immunoprecipitation normalized to anti-Flag immunoblot band intensity from anti-Flag immunoprecipitation. n = 3. ND, normal diet. HSD, high-sucrose diet. Data are presented as mean ± SD. Two-way ANOVA with Sidak's multiple

comparison test was used (**a**, **b**, **d**, **e**, **f**, and **h**). Mantel-Cox test was used (**c** and **g**). One-way ANOVA with Tukey's multiple comparison test was used (**j'**). *p < 0.05. **p < 0.01. ****p < 0.0001. ns, no significant.

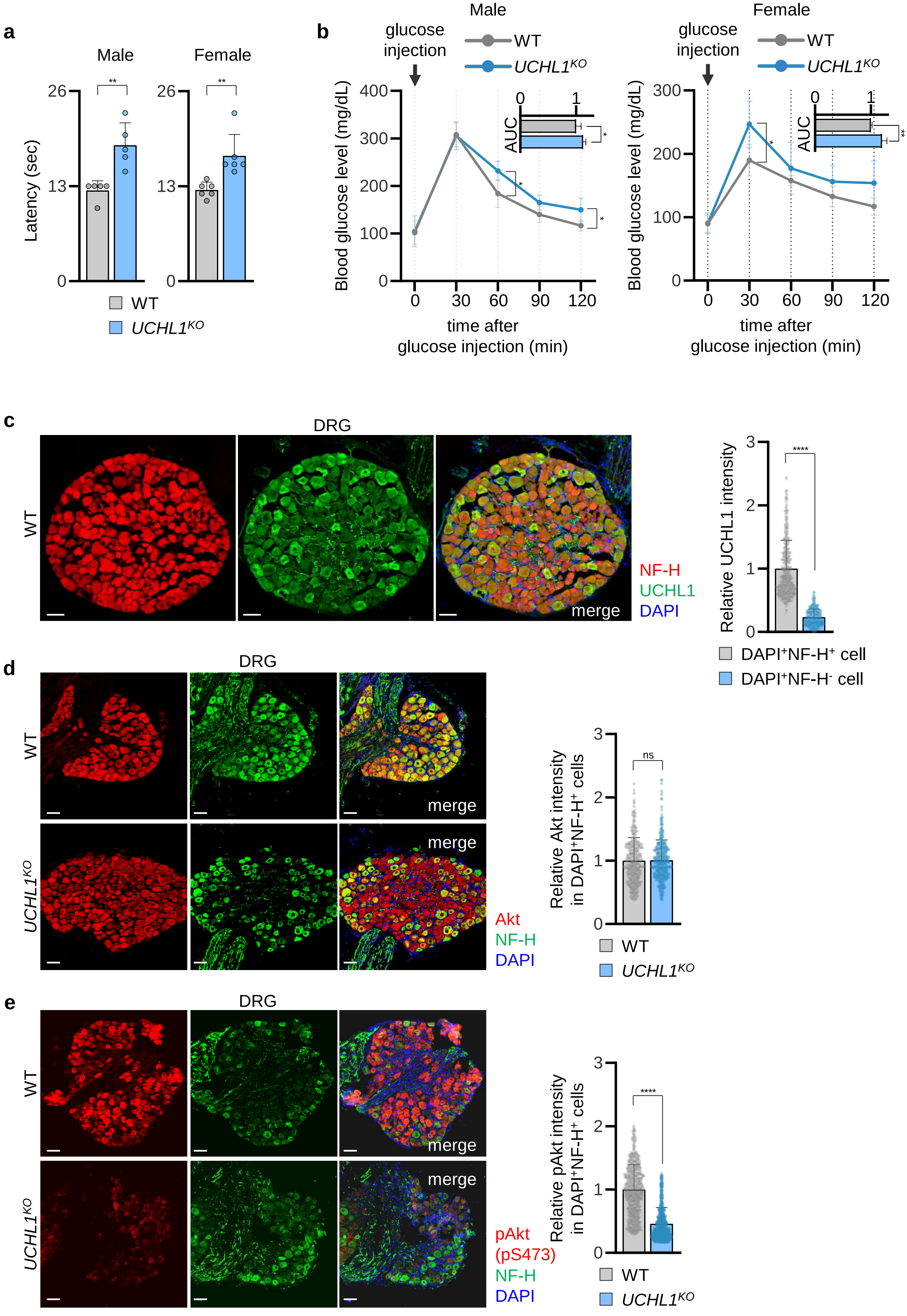
a
Male
Female
Latency (sec)
26
13
0
**
WT
UCHL1KO
b
glucose injection
Male
Female
WT
UCHL1KO
Blood glucose level (mg/dL)
AUC
time after glucose injection (min)
0 30 60 90 120
400
300
200
100
c
DRG
WT
merge
NF-H
UCHL1
DAPI
Relative UCHL1 intensity
****
DAPI+NF-H+ cell
DAPI+NF-H- cell
d
DRG
Akt
NF-H
DAPI
Relative Akt intensity in DAPI+NF-H+ cells
ns
e
pAkt (pS473)
Relative pAkt intensity in DAPI+NF-H+ cells

Supplementary Fig. 12 | *UCHL1* knockout mice also show T2D- and DSN-like phenotypes. **a,** Measuring the latency of first paw pain response in 5-week-old male mice (left) or female mice (right) of WT and $UCHL1^{KO}$. n = 5 (male), 6 (female). **b,** Left, glucose tolerance test (GTT) of 5-week-old male mice of WT and $UCHL1^{KO}$. Glucose was injected at a dosage of 2 mg per gram of body weight, and blood glucose levels were measured before the injection and at 30, 60, 90, and 120 minutes after glucose injection. The bar graph at the upper side represents area under curve (AUC) of the line graph in the same panel. n = 4. Right, GTT of 5-week-old female mice of WT and $UCHL1^{KO}$. Glucose was injected at a dosage of 2 mg per gram of body weight, and blood glucose levels were measured before the injection and at 30, 60, 90, and 120 minutes after glucose injection. The bar graph at the upper side represents AUC of the line graph in the same panel. n = 4. **c,** Left, confocal immunofluorescence images of sectioned DRG from WT (C57BL/6J) mice. Red, NF-H. Green, UCHL1. Blue, DAPI. Scale bar, 50 μm. Right, the relative fluorescence intensity of UCHL1 (green) staining between $DAPI^+NF\text{-}H^+$ and $DAPI^+NF\text{-}H^-$ cells from DRG samples. n = 495 $DAPI^+NF\text{-}H^+$ cells, 395 $DAPI^+NF\text{-}H^-$ cells from 5 mice. **d,** Left, confocal immunofluorescence images of sectioned DRG from WT or $UCHL1^{KO}$ mice. Red, Akt. Green, NF-H. Blue, DAPI. Scale bar, 50 μm. Right, the relative fluorescence intensity of Akt (red) staining in $DAPI^+NF\text{-}H^+$ cell between WT and $UCHL1^{KO}$ mice. n = 528 $DAPI^+NF\text{-}H^+$ cells from 4 WT mice, 702 $DAPI^+NF\text{-}H^+$ cells from 5 $UCHL1^{KO}$ mice. **e,** Left, confocal immunofluorescence images of sectioned DRG from WT or $UCHL1^{KO}$ mice. Red, pAkt. Green, NF-H. Blue, DAPI. Scale bar, 50 μm. Right, the relative fluorescence intensity of pAkt (red) staining in $DAPI^+NF\text{-}H^+$ cell between WT and $UCHL1^{KO}$ mice. n = 851 $DAPI^+NF\text{-}H^+$ cells from 7 WT mice, 1164 $DAPI^+NF\text{-}H^+$ cells from 8 $UCHL1^{KO}$ mice. Two-tailed paired Student's t-test was used (**a**, **b**, **c**, **d**, and **e**). *$p < 0.05$, **$p < 0.01$, ****$p < 0.0001$. ns, no significant.

Name	Sequence
Drosophila DILP1 Forward	5'-TTTAGCCAGCACAACGGT-3'
Drosophila DILP1 Reverse	5'-ATTTCGGTAGACAGTAGATGG-3'
Drosophila DILP2 Forward	5'-GGGGGTGTACTCAATTCCCT-3'
Drosophila DILP2 Reverse	5'-TGGTTGGTTTGGAACCAGAT-3'
Drosophila DILP3 Forward	5'-CGTGAACTTCAATCAGATCG-3'
Drosophila DILP3 Reverse	5'-CTTGGCAGCACAATATCTCA-3'
Drosophila DILP4 Forward	5'-GGCACTGGATGTGATTTGTG-3'
Drosophila DILP4 Reverse	5'-ATCAGGCGCAGTAGTCCAGT-3'
Drosophila DILP5 Forward	5'-CTATTCGATTATGAGGACCAC-3'
Drosophila DILP5 Reverse	5'-GAGTCGCAGTATGCCCTC-3'
Drosophila DILP6 Forward	5'-AGTGCCGACGTCCAAAG-3'
Drosophila DILP6 Reverse	5'-GTTCCGCTTACGCATATCT-3'
Drosophila DILP7 Forward	5'-CCAATCGGACTGGGAGAAC-3'
Drosophila DILP7 Reverse	5'-GTGGTTGTCTTCTTGATCCAA-3'
Drosophila UCH Forward	5'-ACATTGACCGCGGAGTAC-3'
Drosophila UCH Reverse	5'-CGCGGTCAAGGCCAAAA-3'
Drosophila CUL1 Forward	5'-GAGCACGAGAAGTCCCTCAC-3'
Drosophila CUL1 Reverse	5'-AGCTCCACGTAGCACTCGAT-3'
Drosophila shotgun Forward	5'-CGCCGCTAGAGAGTATGTCC-3'
Drosophila shotgun Reverse	5'-CTGGACGATGCTGTATCGAA-3'
Drosophila Rho1 Forward	5'-ATCCCAACACAATTCGGGTA-3'
Drosophila Rho1 Reverse	5'-TGGTTGTTGTTCAGCTCGTT-3'
Drosophila MMP1 Forward	5'-TGGCCGCTCACGAGTT-3'
Drosophila MMP1 Reverse	5'-GAAGCCCTCGCTGATTTC-3'
Drosophila rp49 Forward	5'-AGCTTCAAGATGACCATCCG-3'
Drosophila rp49 Reverse	5'-CCAGGAACTTCTTGAATCCG-3'
Human *CUL1* Forward	5'-CCCAATCATCCAGTAAATCCC-3'
Human *CUL1* Reverse	5'-GTGTGTCTTCTAGTTCTGCC-3'
Human *rp30* Forward	5'-TTCTCGCTAACAACTGCCCA-3'
Human *rp30* Reverse	5'-TGCCACTGTAGTGATGGACAC-3'

Supplementary Table 1 | Primer sequences for real-time PCR

**Name**	**Sequence (AS/AA)**	
Human *SNAI1*	GUGAGUAAUGGCUGUCACU=tt	AGUGACAGCCAUUACUCAC=tt
Human *SNAI2*	GAGAGAAUAAAAGACAGUA=tt	UACUGUCUUUUAUUCUCUC=tt
Human *SNAI3*	GCUGCCUAGUUCCAUUGCA=tt	UGCAAUGGAACUAGGCAGC=tt
Human *CUL1*	GACGAAGGACGAAAAGGAA=tt	UUCCUUUUCGUCCUUCGUC=tt
Human *UCHL1*	GACCUUGGAUGUGGUUUAA=tt	UUAAACCACAUCCAAGGUC=tt
Mouse *UCHL1*	GAAGUUAGCCCUAAAGUUU=tt	AAACUUUAGGGCUAACUUC=tt

Supplementary Table 2 | siRNA sequences
