# Supplementary material for: Diabetic sensory neuropathy and insulin resistance are induced by loss of UCHL1 in Drosophila
Source: Nat Commun. 2024 Jan 11;15:468. doi: 10.1038/s41467-024-44747-9 (PMC10784524; doi:10.1038/s41467-024-44747-9)
Supplement: Supplementary file 3 — Reporting Summary [file 41467_2024_44747_MOESM3_ESM.pdf]

nature portfolio

Corresponding author(s): Jongkyeong Chung

Last updated by author(s): Nov 13, 2023

Reporting Summary

Nature Portfolio wishes to improve the reproducibility of the work that we publish. This form provides structure for consistency and transparency in reporting. For further information on Nature Portfolio policies, see our Editorial Policies and the Editorial Policy Checklist.

Statistics

For all statistical analyses, confirm that the following items are present in the figure legend, table legend, main text, or Methods section.

n/a	Confirmed	
☐	☒	The exact sample size (*n*) for each experimental group/condition, given as a discrete number and unit of measurement
☐	☒	A statement on whether measurements were taken from distinct samples or whether the same sample was measured repeatedly
☐	☒	The statistical test(s) used AND whether they are one- or two-sided<br>*Only common tests should be described solely by name; describe more complex techniques in the Methods section.*
☒	☐	A description of all covariates tested
☐	☒	A description of any assumptions or corrections, such as tests of normality and adjustment for multiple comparisons
☐	☒	A full description of the statistical parameters including central tendency (e.g. means) or other basic estimates (e.g. regression coefficient) AND variation (e.g. standard deviation) or associated estimates of uncertainty (e.g. confidence intervals)
☐	☒	For null hypothesis testing, the test statistic (e.g. *F*, *t*, *r*) with confidence intervals, effect sizes, degrees of freedom and *P* value noted<br>*Give P values as exact values whenever suitable.*
☒	☐	For Bayesian analysis, information on the choice of priors and Markov chain Monte Carlo settings
☒	☐	For hierarchical and complex designs, identification of the appropriate level for tests and full reporting of outcomes
☒	☐	Estimates of effect sizes (e.g. Cohen's *d*, Pearson's *r*), indicating how they were calculated

Our web collection on statistics for biologists contains articles on many of the points above.

Software and code

Policy information about availability of computer code

Data collection	Images were obtained using Carl Zeiss LSM710 confocal microscope (fly experiments) and Yokogawa CSU-W1 SoRa spinning confocal microscope (mouse experiments).<br>Absorbance at specific wavelength was measured using TECAN Infinite M200 pro.<br>Gene expression was measured using Bio-Rad C1000 thermal cycler.<br>Immunoblot images were obtained using LAS 4000.<br>Fly movement videos were recorded using Logitech webcam C910.<br>Nucleus sorting was performed using BD science FACS Aria III.
Data analysis	Fiji v.2.13.1 was used to analyze images.<br>Images obtained from confocal microscopes were processed by ZEN 3.1 blue.<br>Immunoblot images obtained from LAS 4000 were processed by Multi gauge v3.0.<br>Graphpad Prism v.10.1.0 was used to perform statistical analysis.<br>ColabFold was used to predict interactions between proteins.<br>JASPAR was used to predict transcription factors.<br>Protein sequence alignment between fly and human was performed using Clustal Omega.<br>Fly behaviors were analyzed using Ctrax, MATLAB R2020b, and SCAMP.

For manuscripts utilizing custom algorithms or software that are central to the research but not yet described in published literature, software must be made available to editors and reviewers. We strongly encourage code deposition in a community repository (e.g. GitHub). See the Nature Portfolio guidelines for submitting code & software for further information.

Data

Policy information about availability of data

All manuscripts must include a data availability statement. This statement should provide the following information, where applicable:

- Accession codes, unique identifiers, or web links for publicly available datasets
- A description of any restrictions on data availability
- For clinical datasets or third party data, please ensure that the statement adheres to our policy

JASPAR databases are available at https://jaspar.genereg.net. ColabFold databases are available at https://colabfold.mmseqs.com. Clustal Omega for protein sequence alignment is available at https://www.ebi.ac.uk/Tools/msa/clustalo/. All data needed to evaluate the conclusions in the paper are present in the paper and the supplementary information. The raw data and p values for all Figures and Supplementary Figures are available in Source Data files, accompanying this paper.

Research involving human participants, their data, or biological material

Policy information about studies with human participants or human data. See also policy information about sex, gender (identity/presentation), and sexual orientation and race, ethnicity and racism.

Reporting on sex and gender	n/a
Reporting on race, ethnicity, or other socially relevant groupings	n/a
Population characteristics	n/a
Recruitment	n/a
Ethics oversight	n/a

Note that full information on the approval of the study protocol must also be provided in the manuscript.

Field-specific reporting

Please select the one below that is the best fit for your research. If you are not sure, read the appropriate sections before making your selection.

☒ Life sciences ☐ Behavioural & social sciences ☐ Ecological, evolutionary & environmental sciences

For a reference copy of the document with all sections, see nature.com/documents/nr-reporting-summary-flat.pdf

Life sciences study design

All studies must disclose on these points even when the disclosure is negative.

Sample size	Sample size was determined based on standards for experimental cell biology and Drosophila studies, attempting to have a minimum of n = 3 biological replicates with sufficient reproducibility. All experiments were performed at least three independent repeats except for particular cases. Supplementary figure 7e and 7f were performed once due to the fact that these experiments were a screening, of which a mutant with the positive results were confirmed in other experiments. Similarly, supplementary figure 5k, 5m and 11i were conducted one time because the result was verified in other experiments.<br><br>The sample size selection for both in vitro and in vivo experiments was based on previous studies.<br>The following references were used as a basis for determining the sample size:<br>1. Drosophila experiments: Elevated levels of the reactive metabolite methylglyoxal recapitulate progression of type 2 diabetes. Cell Metabolism 27, 926-934 (2018).<br>2. Cell experiments: Loss of UCHL1 rescues the defects related to Parkinson's disease by suppressing glycolysis. Science Advances 7 (2021).<br>3. Mouse experiments: The role of PTEN in primary sensory neurons in processing itch and thermal information in mice. Cell Reports 39 (2022). GTTs and ITTs in mice: simple tests, complex answers. Nature Metabolism 3, 883-886 (2021). Methods used to evaluate pain behaviors in rodents. Frontiers in Molecular Neuroscience 10 (2017).
Data exclusions	No data were excluded from the analysis.
Replication	Each experiment was repeated independently at least three times, and all attempts at replication was successful.
Randomization	Samples from fruit flies and mice were selected at random from the offspring of crosses and assigned to experimental groups based on their genotypes. Samples were allocated randomly for mammalian cell cultures and their analysis.
Blinding	Blinding was performed in all experiments of this study. During data analysis, the investigators were unaware of the specific details or group assignments for blinding.

Reporting for specific materials, systems and methods

We require information from authors about some types of materials, experimental systems and methods used in many studies. Here, indicate whether each material, system or method listed is relevant to your study. If you are not sure if a list item applies to your research, read the appropriate section before selecting a response.

Materials & experimental systems

n/a	Involved in the study
☐	☒ Antibodies
☐	☒ Eukaryotic cell lines
☒	☐ Palaeontology and archaeology
☐	☒ Animals and other organisms
☒	☐ Clinical data
☒	☐ Dual use research of concern
☒	☐ Plants

Methods

n/a	Involved in the study
☒	☐ ChIP-seq
☒	☐ Flow cytometry
☒	☐ MRI-based neuroimaging

Antibodies

Antibodies used

Primary antibody, IHC pAkt (S505), Cell Signaling Technology #4054S, polyclonal, 1:200
Primary antibody, IHC DILP2, gifted from Dr. Yu, polyclonal, 1:200
Primary antibody, IHC Akt, Cell Signaling Technology #4685S, monoclonal (11E7), 1:400
Primary antibody, IHC NF-H, MilliporeSigma # AB5539, polyclonal, 1:1000
Primary antibody, IP Myc, MBL, #M192-3, monoclonal (My3), 1:1,000
Primary antibody, IP Flag, MBL, #M185-3L, monoclonal (FLA-1), 1:1,000
Primary antibody, IHC/western blot pAkt (S473), Cell Signaling Technology #4060S, monoclonal (D9E), 1:1000 (cell experiments), 1:400 (mouse experiments)
Primary antibody, western blot Akt, Cell Signaling Technology #9272S, polyclonal, 1:1000
Primary antibody, western blot Flag, Cell Signaling Technology #2368, polyclonal, 1:1,000
Primary antibody, IHC/western blot UCHL1, Cell Signaling Technology #13179S, monoclonal (D3T2E), 1:1000 (cell experiments), 1:200 (mouse experiments)
Primary antibody, western blot Tubulin, DSHB, monoclonal (E7), 1:5,000
Primary antibody, western blot HA, Cell Signaling Technology #3724, monoclonal (C29F4), 1:1,000
Primary antibody, western blot IRS1, Cell Signaling Technology #2390S, monoclonal (59G8), 1:1000
Primary antibody, western blot CUL1, Cell Signaling Technology #4995S, polyclonal, 1:1,000
Primary antibody, western blot NEDD8, Cell Signaling Technology #2745, polyclonal, 1:1,000
Secondary antibody, western blot HRP-mouse, Jackson ImmunoResearch #115-035-146, polyclonal, 1:5,000
Secondary antibody, western blot HRP-rabbit, Jackson ImmunoResearch #111-035-144, polyclonal, 1:5,000
Secondary antibody, IHC TRITC-rabbit, Jackson ImmunoResearch #111-296-144, polyclonal, 1:200 (fly experiments), 1:400 (mouse experiments)
Secondary antibody, IHC Cy3-rabbit, Jackson ImmunoResearch #711-165-152, polyclonal, 1:400
Secondary antibody, IHC Alexa Fluor 488-chicken, Jackson ImmunoResearch #703-545-155, polyclonal, 1:400

Validation

Primary antibody, IHC pAkt (S505), Cell Signaling Technology #4054S: 160 citations reported on manufacturer's website (https://www.cellsignal.com/product/productDetail.jsp?productId=4054)
Primary antibody, IHC DILP2, Drosophila Adiponectin Receptor in Insulin Producing Cells Regulates Glucose and Lipid Metabolism by Controlling Insulin Secretion, PLoS ONE, 2013 (doi: 10.1371/journal.pone.0068641)
Primary antibody, IHC Akt , Cell Signaling Technology #4685S: 1855 citations reported on manufacturer's website (https://www.cellsignal.com/products/primary-antibodies/akt-pan-11e7-rabbit-mab/4685)
Primary antibody, IHC NF-H, MilliporeSigma # AB5539: 1855 citations reported on manufacturer's website (https://www.merckmillipore.com/KR/ko/product/Anti-Neurofilament-H-Antibody,MM_NF-AB5539#anchor_REF)
Primary antibody, IP Myc, MBL, #M192-3: 24 citations reported on manufacturer's website (https://www.mblbio.com/bio/g/dtl/A/?pcd=M192-3#u-pub)
Primary antibody, IP Flag, MBL, #M185-3L: 73 24 citations reported on manufacturer's website (https://www.mblbio.com/bio/g/dtl/A/?pcd=M185-3L)
Primary antibody, western blot pAkt (S473), Cell Signaling Technology #4060S: 10229 citations reported on manufacturer's website (https://www.cellsignal.com/products/primary-antibodies/phospho-akt-ser473-d9e-xp-rabbit-mab/4060)
Primary antibody, western blot Akt, Cell Signaling Technology #9272S: 10043 citations reported on manufacturer's website (https://www.cellsignal.com/products/primary-antibodies/akt-antibody/9272)
Primary antibody, western blot Flag, Cell Signaling Technology #2368: 762 citations reported on manufacturer's website (https://www.cellsignal.com/products/primary-antibodies/dykddddk-tag-antibody-binds-to-same-epitope-as-sigma-aldrich-anti-flag-m2-antibody/2368)
Primary antibody, western blot UCHL1, Cell Signaling Technology #13179S: 32 citations reported on manufacturer's website (https://www.cellsignal.com/products/primary-antibodies/uchl1-d3t2e-xp-rabbit-mab/13179)
Primary antibody, western blot Tubulin, DSHB, monoclonal (E7): 130 references reported on manufacturer's website (https://dshb.biology.uiowa.edu/E7_2)

Primary antibody, western blot HA, Cell Signaling Technology #3724: 2665 citations reported on manufacturer's website (https://www.cellsignal.com/products/primary-antibodies/ha-tag-c29f4-rabbit-mab/3724)
Primary antibody, western blot IRS1, Cell Signaling Technology #2390S: 59 citations reported on manufacturer's website (https://www.cellsignal.com/products/primary-antibodies/irs-1-59g8-rabbit-mab/2390)
Primary antibody, western blot CUL1, Cell Signaling Technology #4995S: 29 citations reported on manufacturer's website (https://www.cellsignal.com/products/primary-antibodies/cul1-antibody/4995)
Primary antibody, western blot NEDD8, Cell Signaling Technology #2745: 38 citations reported on manufacturer's website (https://www.cellsignal.com/products/primary-antibodies/nedd8-antibody/2745)
Secondary antibody, western blot HRP-mouse, Jackson ImmunoResearch #115-035-146: 634 citations reported on manufacturer's website (https://www.jacksonimmuno.com/catalog/products/115-035-146)
Secondary antibody, western blot HRP-rabbit, Jackson ImmunoResearch #111-035-144: 1511 citations reported on manufacturer's website (https://www.jacksonimmuno.com/catalog/products/111-035-144)
Secondary antibody, IHC Cy3-rabbit, Jackson ImmunoResearch #711-165-152: 1780 citations reported on manufacturer's website (https://www.jacksonimmuno.com/catalog/products/711-165-152)
Secondary antibody, IHC Alexa Fluor 488- chicken, Jackson ImmunoResearch #703-545-155: 871 citations reported on manufacturer's website (https://www.jacksonimmuno.com/catalog/products/703-545-155)

Eukaryotic cell lines

Policy information about cell lines and Sex and Gender in Research

Cell line source(s)	Human Embryonic Kidney 293E (HEK293E) and Mouse Embryonic Fibroblast (MEF) cell lines were gifted from Dr. John Blenis at Cornell University.<br>SH-SY5Y and SNU398 cell lines were obtained from Korean Cell Line Bank (Commercial).<br>UCHL1 KO HEK293E and UCHL1 KO SH-SY5Y were generated by Eunju Yoon and Su Jin Ham, respectively.
Authentication	Cell lines were authenticated using morphology and immunoblot analyses, and were carefully labeled and tracked.
Mycoplasma contamination	Cells used for all experiments were mycoplasma negative.
Commonly misidentified lines<br>(See ICLAC register)	No commonly misidentified cell lines were used in this study.

Animals and other research organisms

Policy information about studies involving animals; ARRIVE guidelines recommended for reporting animal research, and Sex and Gender in Research

Laboratory animals	3-day-old and 30-day-old flies, clearly defined in the figure legends, were used for this study, with the exception of the TUNEL assay. For the TUNEL assay, 20-day-old flies were used.<br>Fly strains used in this study:<br>w1118, cg-GAL4, mef2-GAL4, elav-GAL4, nSyb-GAL4, DILP2-GAL4, OK371-GAL4, hs-GAL4, OK6-GAL4, tub-GAL80TS, UAS-DILP2, UAS-nlsGFP, UAS-IRCA, UAS-PI3KCAAX, UAS-myrAkt, UAS-laminGFP, UAS-GFP, UAS-UCH RNAi, UAS-TRAF4 RNAi, UAS-Snail RNAi, UAS-UBA3 RNAi, UAS-UBE2M RNAi, UAS-RBX1 RNAi, UAS-CUL3 RNAi, UAS-CUL5 RNAi, UAS-CUL1, UAS-Snail, UAS-GSK3BCA, UAS-GSK3BDN UAS-Cbl RNAi, UAS-CUL1 RNAi, UAS-Drice RNAi, UAS-IRS1 RNAi, UAS-CCND RNAi, UAS-CCNE RNAi, UAS-CDK4 RNAi, UAS-Wee1 RNAi, UAS-DILP2 RNAi, UAS-Akt RNAi, UAS-dIRS1, UAS-DILP2-Flag, UAS-PKM, UCHKO, UCHC93S , UAS-PKM, UAS-UCH<br><br>Mouse strains used in this study:<br>5-week-old male and female mice of C57BL/6J and Uchl1(gad-j)/(gad-j) strains were used.
Wild animals	No wild animals were used.
Reporting on sex	Fly experiments were conducted on both male and female flies, with the exception of imaging sensory nuclei/neurons. The imaging of sensory nuclei/neurons was specifically performed on male flies due to the presence of the sex comb, which is unique to males. By mounting the legs of the fruit flies in a manner that made the sex comb visible, we were able to capture high-quality images. All mouse experiments were conducted on both male and female mice.
Field-collected samples	No field-collected samples were involved in this study.
Ethics oversight	No ethical approval was required for fly experiments according to Institutional Animal Care and Use Committee in Seoul National University. All mouse experiments were approved by the Institutional Animal Care and Use Committee at Pohang University of Science and Technology and performed in accordance with its guidelines (POSTECH-2022-0080).

Note that full information on the approval of the study protocol must also be provided in the manuscript.
